# Rad52 Sumoylation Prevents the Toxicity of Unproductive Rad51 Filaments Independently of the Anti-Recombinase Srs2

**DOI:** 10.1371/journal.pgen.1003833

**Published:** 2013-10-10

**Authors:** Aline Esta, Emilie Ma, Pauline Dupaigne, Laurent Maloisel, Raphaël Guerois, Eric Le Cam, Xavier Veaute, Eric Coïc

**Affiliations:** 1 CEA, DSV, iRCM, SIGRR, LRGM, Fontenay-aux-Roses, France; 2 Laboratoire de Microscopie Moléculaire et Cellulaire, UMR 8126, Interactions Moléculaires et Cancer, CNRS–Université Paris Sud–Institut de Cancérologie Gustave Roussy, Villejuif, France; 3 CEA, DSV, iBiTecS, LBSR, Gif-sur-Yvette, France; National Cancer Institute, United States of America

## Abstract

The budding yeast Srs2 is the archetype of helicases that regulate several aspects of homologous recombination (HR) to maintain genomic stability. Srs2 inhibits HR at replication forks and prevents high frequencies of crossing-over. Additionally, sensitivity to DNA damage and synthetic lethality with replication and recombination mutants are phenotypes that can only be attributed to another role of Srs2: the elimination of lethal intermediates formed by recombination proteins. To shed light on these intermediates, we searched for mutations that bypass the requirement of Srs2 in DNA repair without affecting HR. Remarkably, we isolated *rad52-L264P*, a novel allele of *RAD52*, a gene that encodes one of the most central recombination proteins in yeast. This mutation suppresses a broad spectrum of *srs2*Δ phenotypes in haploid cells, such as UV and γ-ray sensitivities as well as synthetic lethality with replication and recombination mutants, while it does not significantly affect Rad52 functions in HR and DNA repair. Extensive analysis of the genetic interactions between *rad52-L264P* and *srs2*Δ shows that *rad52-L264P* bypasses the requirement for Srs2 specifically for the prevention of toxic Rad51 filaments. Conversely, this Rad52 mutant cannot restore viability of *srs2*Δ cells that accumulate intertwined recombination intermediates which are normally processed by Srs2 post-synaptic functions. The avoidance of toxic Rad51 filaments by Rad52-L264P can be explained by a modification of its Rad51 filament mediator activity, as indicated by Chromatin immunoprecipitation and biochemical analysis. Remarkably, sensitivity to DNA damage of *srs2*Δ cells can also be overcome by stimulating Rad52 sumoylation through overexpression of the sumo-ligase *SIZ2*, or by replacing Rad52 by a Rad52-SUMO fusion protein. We propose that, like the *rad52-L264P* mutation, sumoylation modifies Rad52 activity thereby changing the properties of Rad51 filaments. This conclusion is strengthened by the finding that Rad52 is often associated with complete Rad51 filaments *in vitro*.

## Author Summary

Homologous recombination (HR) is essential for double-strand break repair and participates in post-replication restart of stalled and collapsed replication forks. However, HR can lead to genome rearrangements and has to be strictly controlled. The budding yeast Srs2 is involved in the prevention of high crossing-over frequencies and in the inhibition of HR at replication forks. Nevertheless, important phenotypes of *srs2*Δ mutants, like sensitivity to DNA damage and synthetic lethality with replication and recombination mutants, can only be attributed to another role of Srs2: the elimination of lethal intermediates formed by recombination proteins. The nature of these intermediates remains to be defined. In a screen designed to uncover mutations able to suppress *srs2*Δ phenotypes, we isolated a novel allele of Rad52 (*rad52-L264P*), the gene that codes for the major Rad51 nucleoprotein filament mediator. Interestingly, we observed that *rad52-L264P* bypasses the requirement for Srs2 without affecting DNA repair by HR. We also found that Rad52-L264P specifically prevents the formation of unproductive Rad51 filaments before strand invasion, allowing us to define Srs2 substrates. Further analysis showed that Rad52-L264P mimics the properties of the Rad52-SUMO conjugate, revealing that Rad52 assembles Rad51 filaments differently according to its sumoylation status.

## Introduction

Homologous recombination (HR) is fundamental for the repair of DNA double-strand breaks (DSBs). It is also involved in the error-free fill-in of single-strand gaps generated by replication fork stalling or incomplete DNA repair. Defects in HR are associated with many cancers, both hereditary and sporadic [1], which underlines the essential nature of this process. The mechanisms and proteins involved in HR have been well conserved throughout evolution and much of our knowledge on HR comes from studies conducted with the yeast *Saccharomyces cerevisiae* (*S. cerevisiae*) (reviewed in [2], [3]). HR involves the interaction of a 3′-single stranded DNA (ssDNA) end with a homologous double-strand DNA (dsDNA) molecule, which is used as a template for DNA synthesis. In eukaryotes, the recombinase Rad51 forms a nucleoprotein filament on the ssDNA which undergoes synapsis and strand invasion of the homologous duplex DNA to form a stable D-loop (reviewed in [4]). However, the presence of replication protein A (RPA) previously bound to ssDNA prevents Rad51-mediated strand exchange *in vitro*. This inhibition is overcome by the addition of Rad52 or the Rad55-Rad57 heterodimer (the Rad51 paralogs of *S. cerevisiae*), defining these proteins as Rad51 filament mediators (reviewed in [4]).

Rad52 exhibits the greatest Rad51 filament mediator activity in *S. cerevisiae*. Via its interaction with both RPA and Rad51, it stimulates the removal of RPA and recruits Rad51 to DNA (reviewed in [4]). This mediator function is highlighted by the severe phenotypes caused by null mutations of the *RAD52* gene: γ-ray sensitivity and highly reduced levels of both mitotic and meiotic HR (reviewed in [3]). The Rad52 protein also has the capacity to anneal homologous ssDNA *in vitro* [5]. This activity is involved in Rad51-independent single-strand annealing (SSA) [6] and possibly in the capture of the second end of a DSB to generate double Holliday junction (dHJ) intermediates [7]–[9].

The *S. cerevisiae* Rad52 protein is subject to post-translational modifications but it is unclear how these modulate Rad52 activities. Rad52 is constitutively phosphorylated throughout the cell cycle on some serine and/or threonine residues and additional phosphorylations are induced specifically in S phase [10]. The phosphorylated residues have not yet been identified. Rad52 also undergoes sumoylation at lysines 10, 11 and 220 after exposure to DNA-damaging agents that induce DSBs. This modification depends on the SUMO-conjugating enzyme Ubc9 and on the SUMO-ligase Siz2 [11]. ssDNA accumulation is necessary for sumoylation of Rad52 [11], [12]. It has been reported that loss of Rad52 sumoylation decreases protein stability without significantly affecting HR levels or recruitment of Rad52 to DNA damage [11], [13]. Sumoylation appears to facilitate the exclusion of Rad52 recombination foci from the nucleolus to maintain a low level of recombinational repair at the ribosomal gene locus [14]. Recently, it was shown that Rad52, RPA and Rad59 are modified by a sumoylation wave leading to simultaneous multisite modification. Catalyzed by a DNA-bound SUMO ligase, this wave stabilizes physical interactions between the proteins [15]. However, Rad52 sumoylation might also restrict Rad51 filament formation through the SUMO-targeted Cdc48 segregase that can curb Rad52-Rad51 physical interaction and displace these proteins from DNA [16]. How exactly phosphorylation and sumoylation of Rad52 affect Rad51 filament formation remains to be determined.

Contrasting with its primordial role in DNA repair, HR may lead to potentially lethal intermediates. This was first revealed by the study of the Srs2 helicase, a major actor in the avoidance of such intermediates (reviewed in [17]). UV sensitivity of *srs2*Δ cells is suppressed by the ablation of the Rad51 protein [18], which suggests that Srs2 is required for the elimination of toxic UV-induced recombination structures. Furthermore, it was shown that leaky alleles of *RAD51* or *RAD52*, which form abortive recombination intermediates, trigger Srs2 activity [19], [20]. Finally, negative interactions between *srs2*Δ and genes involved in DNA replication or recombination, such as *sgs1*Δ, *rad54*Δ or *mrc1*Δ, are rescued by mutations preventing or altering HR (*rad51*Δ, *rad52*Δ, *rad55*Δ and *rad57*Δ) 21–23. Thus, it was concluded that ablation of these genes can also induce the formation of lethal recombination intermediates normally eliminated by Srs2.

All these studies indicate that a key role of Srs2 is to avoid the formation of lethal structures induced by HR. Several studies point out that Rad51 filaments on ssDNA could be the lethal structures eliminated by Srs2. First, *in vitro* experiments show that Srs2 can disrupt Rad51 filaments thanks to its translocase activity [24], [25]. Second, *srs2*Δ strains show a three- to four-fold increase in the number of budded cells that contain a Rad51 or Rad54 focus compared with wild-type (WT) cells [26]. Finally, it has also been reported that some of the *srs2*Δ phenotypes, like the co-lethality with *rad54*Δ or the high sensitivity to a persistent DSB, depend at least partially on the DNA damage checkpoint [27], [28]. Unproductive Rad51 filaments could induce this persistent checkpoint response.

Srs2 is also necessary to complete DSB repair by HR in haploid cells, when the homologous sequence is located on another chromosome. In this context, the low viability of *srs2*Δ cells is associated with a strong increase in the level of crossing-over (CO) associated with gene conversion [29], [30]. This suggests that Srs2 avoids the formation of CO by promoting synthesis-dependent strand annealing [31]. This is supported by recent *in vivo* work showing that Srs2 promotes the formation of non-crossing over products mostly through its helicase activity [32], possibly by dismantling nicked HJs. Additionally, the increased sensitivity to UV and γ-ray irradiation of *srs2*Δ homozygous diploid cells compared with haploids was proposed to be related to the resolution of specific interactions between homologous chromosomes, probably related to HR [33]. Altogether, these data suggest that lethal recombination structures eliminated by Srs2 could also be intertwined recombination intermediates.

To gain insight into the nature of lethal recombination intermediates eliminated by Srs2, we searched for mutants that suppress the sensitivity of *srs2*Δ cells to the radiomimetic DNA alkylating agent methyl methanesulfonate (MMS). This screen was designed to select against mutations in genes that are essential for HR because they generally confer high sensitivity to this drug. Yet we found an allele of *RAD52* (*rad52-L264P*) that can completely suppress sensitivity to MMS in *srs2*Δ cells. Our extensive analysis indicates that *rad52-L264P* suppresses defects in *srs2*Δ cells associated with presynaptic Rad51 filaments rather than with the resolution of recombination intermediates. This suppression is related to a modulation of Rad52 mediator activity. We also show that, like *rad52-L264P*, Rad52 sumoylation leads to the suppression of *srs2*Δ cells defects, suggesting that sumoylation modulates Rad52 mediator activity.

## Results

### A novel allele of *RAD52* bypasses the requirement of *SRS2* for resistance to DNA damage

**Figure 1:**
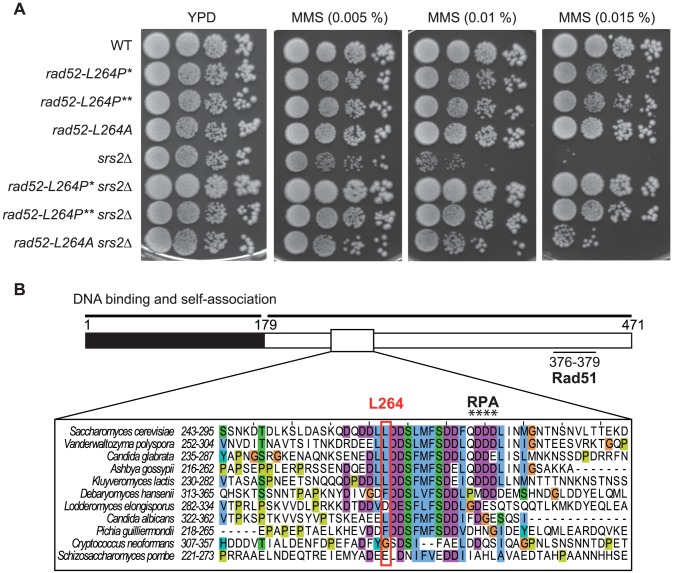
Characterization of *rad52-L264P*, a suppressor of the MMS sensitivity of *srs2*Δ mutants that avoid the formation of toxic recombination intermediates. (A) Serial 10-fold dilutions of haploid strains with the indicated genotypes were plated onto rich media (YPD) containing different MMS concentrations. rad52-L264P* denotes the original isolated mutant strain and rad52-L264P** denotes a strain in which the *RAD52* gene was replaced by the mutant newly generated by directed mutagenesis. (B) Conservation of a motif comprising L264. The primary structure of Rad52 is schematized showing the conserved N-terminus moiety containing the major DNA binding and self-association domains (black, amino acids 1 to 179) as well as the C-terminus part (white, amino acids 180 to 471) containing the RPA (amino acids 275 to 278) and the Rad51 (amino acids 376 to 379) binding domains. The alignment of the Rad52 protein in Hemiascomycetes species shows the conservation of a domain containing the non-essential L264 residue and the QDDD residues essential for RPA binding and consequently for Rad52 mediator activity. The color code used in the alignment follows the default ClustalX color scheme as implemented in JalView (see Material and Methods). Cyan is for fully hydrophobic (I, L, V, M, F), turquoise for aromatic residues containing polar moieties (Y, H), green for small polar (T, S), purple for acidic (D, E), orange for glycine (G) and dark yellow for proline (P) residues.

To characterize factors involved in the formation of toxic recombination intermediates eliminated by Srs2, we selected mutants that suppress the MMS sensitivity of *srs2*Δ haploid cells by plating them on rich medium containing 0.015% MMS. Several well-grown colonies were isolated and backcross analyses showed that the suppressor mutations responsible for MMS resistance were all monogenic. Genetic analyses showed that they affected different genes. We genetically mapped one of these mutations at 25 cM from the *PIF1* gene. Around this position, we found *RAD52* to be a good candidate since it was involved in DNA repair. The sequence of the *RAD52* gene showed a single T to C transversion at position 790 of the open reading frame (ORF), leading to a change of leucine 264 to a proline. To confirm that this mutation is solely responsible for the phenotype, directed mutagenesis was used to create an integrative plasmid which was introduced by gene replacement in a new *srs2*Δ strain using the pop-in pop-out technique. MMS sensitivity suppression was equivalent to that observed in the original suppressed strain (Figure 1A).

Rad52 is composed of a highly conserved N-terminus that forms ring structures [34], [35]. Outside the N-terminus, the protein is less conserved. Sequence alignments show that L264 belongs to a stretch of well-conserved amino acids (positions 261–283) in Hemiascomycetes, located outside the highly conserved N-terminus (Figure 1B). L264 is located ten residues upstream of the QDDD motif essential for RPA binding [36], (Figure 1B). Note that while *rad52-L264P* is only slightly sensitive to MMS (Figure 1A), *rad52-Q275A/D276A/D277A/D278A* is a null allele [36].

We wondered if the change to a proline and not the loss of the leucine itself was significant for the suppression phenotype. Therefore, we replaced the leucine with an alanine by directed mutagenesis. We found that *rad52-L264A* suppresses the MMS sensitivity of *srs2*Δ cells, but not as well as *rad52-L264P* (Figure 1A). Therefore, even if changing the leucine to a proline has a more radical effect, probably because it affects the domain more extensively, it is the leucine ablation that confers the suppression phenotype.

### *rad52-L264P* cells are proficient in HR

**Figure 2:**
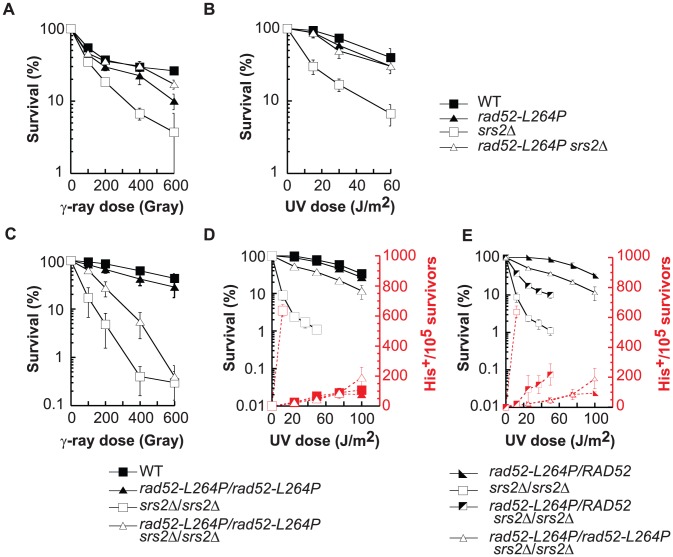
*rad52-L264P* does not affect DNA repair or HR, whereas it completely suppresses *srs2*Δ phenotypes. (A, B) Survival curves of haploid cells grown in log phase culture exposed to γ-ray or UV light. (C, D, E) Survival curves and heteroallelic HR frequency of diploid cells grown in log phase culture exposed to γ-ray or UV light. The results shown are the average of at least 3 independent experiments.

**Figure 9:**
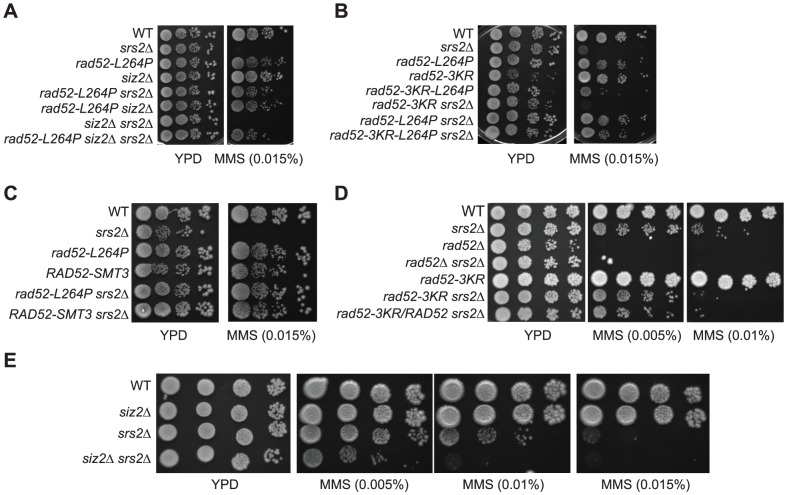
Rad52-L264P behaves like Rad52-SUMO. (A to E) Spot assay of haploid cells with the indicated genotype on rich medium (YPD) containing increasing MMS concentrations. Note that the deletion of the *SIZ2* gene (A) and the *rad52-3KR* allele (B) cannot suppress the MMS sensitivity of *srs2*Δ cells. Therefore, the MMS resistance of *rad52-L264P siz2*Δ *srs2*Δ and *rad52-3KR-L264P srs2*Δ cells is only related to *rad52-L264P*. (D) Note that the haploid strain spotted at the bottom contains both *rad52-3KR* and *RAD52* alleles.

To characterize the effect of the mutation on Rad52 activities, we constructed a *rad52-L264P* single mutant strain. *rad52-L264P* is not sensitive to incubation at 16°C or 37°C (Figure S1A) and, unlike the deletion of *RAD52*, it does not affect the rate of spontaneous mutagenesis in the *CAN1* gene (Figure S1B). The growth rate is also unchanged compared with WT (90 minutes), while the deletion of *RAD52* leads to a significant increase of the doubling time (155 minutes). Surprisingly, cells carrying this mutation are strongly resistant to MMS, while *rad52*Δ makes cells extremely sensitive (Figures 1A and 9D). *rad52-L264P* cell resistance to γ-ray and to UV is also comparable to that of WT cells (Figure 2A and B). UV-induced recombination between *his7-1* and *his7-2* heteroalleles in *rad52-L264P* homozygous diploid cells is indistinguishable from that of WT cells (Figure 2D), suggesting that gene conversion is not affected by this mutation. Altogether, these data show that *rad52-L264P* does not substantially affect the activity of the protein.

### UV irradiation and γ-ray sensitivities as well as the UV-induced hyper-recombination phenotype of *srs2*Δ are suppressed by *rad52-L264P*

*rad52-L264P* completely suppresses *srs2*Δ haploid cell growth defect on MMS plates (Figure 1A). It also suppresses *srs2*Δ γ-ray and UV sensitivities (Figure 2A and B). UV induces mostly single strand gaps, while γ-ray and MMS produce also DSBs. Therefore, the deadly recombination intermediates induced by both kinds of lesions in *srs2*Δ cells are not toxic or are not formed in the *rad52-L264P srs2*Δ background.

*rad52-L264P* also completely suppresses the previously described UV-induced *srs2*Δ hyper-recombination phenotype [33], [37]. Frequencies of UV-induced recombination in diploid cells measured between *his7-1* and *his7-2* heteroalleles are the same in *rad52-L264P srs2*Δ and in WT cells (Figure 2D).

Interestingly, the UV sensitivity and the hyper-recombination phenotype of *srs2*Δ/*srs2*Δ diploid cells were only partially suppressed when *rad52-L264P* and *RAD52* alleles were co-expressed in comparison with homozygous *rad52-L264P* diploids (Figure 2E). Therefore, the WT and *rad52-L264P* alleles of *RAD52* are co-dominant, which implies that *rad52-L264P* cannot be a simple hypomorphic allele. The Rad52-L264P protein mediates the formation of enough functional Rad51 filaments for HR and DNA repair to occur without leading to accumulation of deadly recombination intermediates in *srs2*Δ cells.

### *rad52-L264P* suppresses *srs2*Δ synthetic sickness or lethality with DNA repair and replication mutants

**Figure 3:**
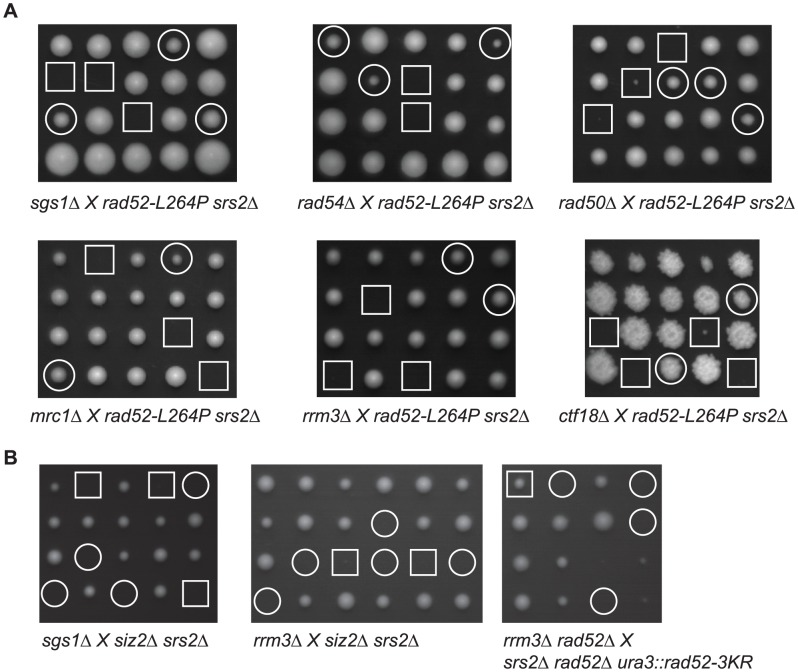
*rad52-L264P* suppresses mutations that are synthetically lethal with *srs2*Δ. (A) Tetrad analysis of crosses between haploid *rad52-L264P srs2*Δ strains and haploid mutants synthetically lethal with *srs2*Δ. Double mutant spores, which do not contain *rad52-L264P*, are indicated by white squares. The white circles mark triple mutants. (B) *siz2*Δ and *rad52-3KR* do not suppress the synthetic lethality of *srs2*Δ *sgs1*Δ and *srs2*Δ *rrm3*Δ mutants. In crosses involving *siz2*Δ, white squares display spores of *srs2*Δ *sgs1*Δ or *srs2*Δ *rrm3*Δ genotypes and white circles indicate triple mutants. To analyze the genetic interaction between *rad52-3KR* inserted at the *URA3* locus and the synthetically lethal *rrm3*Δ *srs2*Δ double mutant, diploids homozygous for *rad52*Δ were sporulated, in order to avoid the co-expression of *RAD52* and *rad52-3KR*. The white square indicates *srs2*Δ *rrm3*Δ *rad52*Δ triple mutants and white circles indicate unviable *srs2*Δ *rrm3*Δ *rad52*Δ *ura3*::*rad52-3KR* monosporic colonies.

Deleting numerous genes involved in DNA replication or recombination can also induce the formation of lethal recombination intermediates in the absence of Srs2. The deletion of those genes reveals negative interactions with *srs2*Δ in a *RAD51*-dependent manner. Some of these genes, like *RAD50* and *RAD54*, are involved in the normal maturation process of recombination intermediates [22], [27], [38]. Another set of genes is involved in DNA replication. Among them are *RRM3* [39], *MRC1* and *CTF18* [23]. The *srs2*Δ mutation is also synthetically lethal with *sgs1*Δ, but Sgs1 is involved in recombination and potentially in replication. Therefore, it is not clear which function of Sgs1 is required to avoid *srs2*Δ death [40]. We wondered if some of these negative interactions would be suppressed by *rad52-L264P*. A *rad52-L264P srs2*Δ strain was crossed with strains containing deletions of the genes interacting negatively with *srs2*Δ (Figure 3A). Interestingly, tetrad analysis showed that all the negative interactions we tested were suppressed by *rad52-L264P*. Triple mutant strains' doubling times ranged from 92 to 147 min (Table S1), indicating that even if barriers to replication might persist in some background, the suppression is rather strong. We conclude from these experiments that there is a feature common to toxic recombination intermediates eliminated by Srs2 in the different recombination and replication mutants as well as in cells exposed to DNA-damaging agents. Therefore, the toxicity of these intermediates disappears in *rad52-L264P srs2*Δ cells or, alternatively, the intermediates themselves are not formed.

### *rad52-L264P* suppresses *srs2*Δ defects associated with presynaptic Rad51 nucleoprotein filament formation

**Figure 4:**
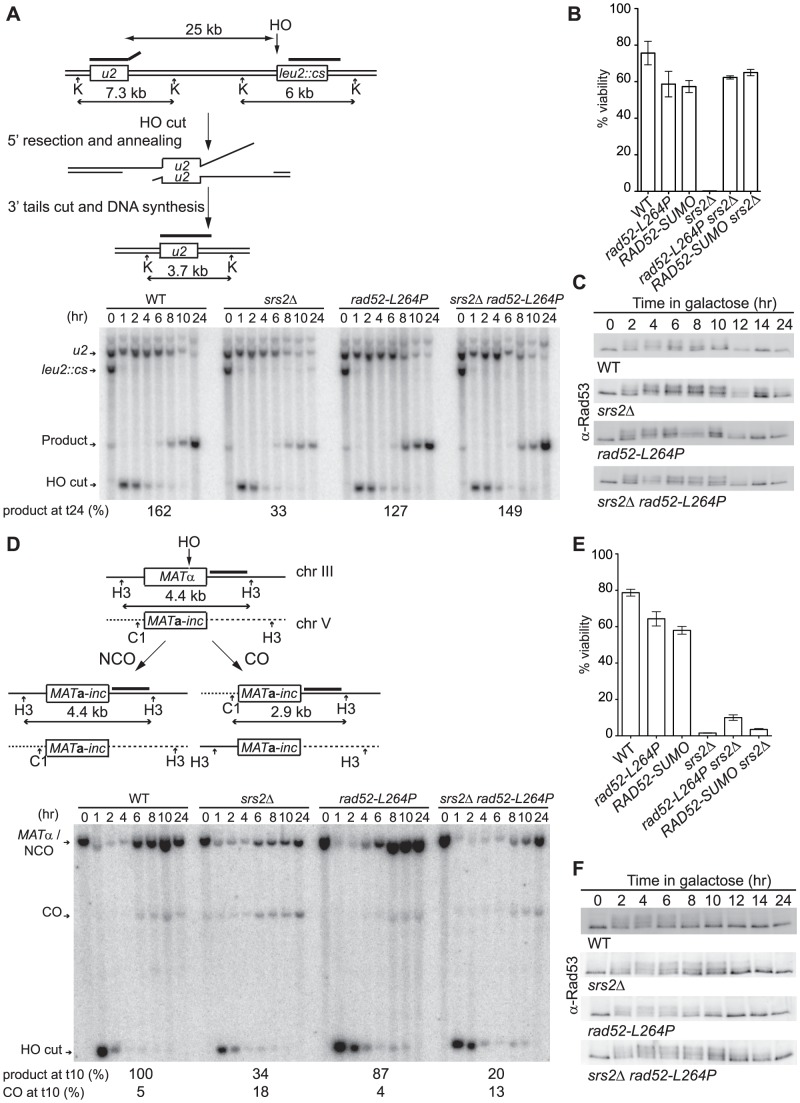
*rad52-L264P* can only suppress *srs2*Δ deficiencies in the management of unproductive Rad51 filaments. Schematic representation and Southern blot analysis of the two HO-induced DSB repair systems involving SSA between two direct repeats 25 kb apart (A) and gene conversion between ectopic copies of *MAT* (D). The kinetic of repair in the SSA system after HO induction by addition of galactose to the medium was monitored by probing a Southern blot of *Kpn*I (K) digested genomic DNA of cells harvested at the indicated time with a PCR fragment complementary to the 3′end of *LEU2* (bold line). Quantification of the product band relative to the parental band (leu2::cs) measured at 24 hours is indicated (see Material and Methods for more information). To follow gene conversion of the *MAT*α allele after DSB induction, DNA was digested with *Cla*I (C1) and *Hind*III (H3), and probed with a *MAT* distal PCR fragment (bold line). The two possible outcomes, gene conversion associated with a CO or not (NCO), are indicated. The proportion of repaired products (NCO+CO) relative to the parental band (*MAT*α) and the proportion of CO among repair products measured at 10 hours are indicated (see Material and Methods for more information). (B and E) Cell viability after DSB formation in both assays. (C and F) Western blot analysis of Rad53 phosphorylation after HO induction in both systems.

The defects associated with *srs2*Δ in haploid cells can be suppressed by *rad52-L264P*. However, resistance to γ-ray and UV irradiation is only partially restored in *srs2*Δ *rad52-L264P* homozygous diploids (Figure 2C and D). It has been proposed that the higher sensitivity of *srs2*Δ homozygous diploids compared with haploids is related to the resolution of specific interactions between homologous chromosomes, probably related to HR [33]. Thus, *rad52-L264P* would not suppress *srs2*Δ deficiency in the resolution of recombination intermediates involving homologous chromosomes. This would also mean that the important role of Srs2 in DNA damage resistance in haploid cells would not be related to the resolution of recombination intermediates. We propose rather that the defect in Srs2 observed in haploid cells is related to the formation of Rad51 filaments that are toxic because they do not achieve strand invasion (because they are deficient or because a homologous sequence is not available to perform strand invasion) and cannot be removed from ssDNA. These filaments can be defined as toxic presynaptic Rad51 filaments. Conversely, the fraction of *srs2*Δ sensitivity that cannot be suppressed by *rad52-L264P* in diploid cells may be related to a “postsynaptic” role of Srs2. To test this hypothesis, we took advantage of two recombination systems allowing the repair of a single DSB created by a galactose-inducible HO endonuclease. Both systems require Srs2 to survive the formation of the DSB. The first one involves SSA between direct repeats located 25 kb apart (Figure 4A) and the other one uses ectopic gene conversion to repair the DSB (Figure 4D) [28], [29]. Srs2 is required in SSA to remove Rad51 accumulating on ssDNA generated from DSB processing [41], while during ectopic gene conversion *srs2*Δ cells fail to properly resolve recombination intermediates [29]. According to our hypothesis, *rad52-L264P* should only suppress the poor viability of *srs2*Δ cells after HO induction in the SSA system. This is exactly what we observed: *rad52-L264P* suppressed *srs2*Δ low cell viability strongly in the SSA system (Figure 4B), but only marginally in the gene conversion ectopic system (Figure 4E). Monitoring of DSB repair in both systems by Southern blot analysis showed that *rad52-L264P* restored a normal level of SSA product formation in *srs2*Δ cells (Figure 4A). However, the gene conversion products in the ectopic system did not accumulate as much as in WT cells (Figure 4D). Note that the survival rates after DSB induction in the SSA and the ectopic systems were largely unchanged in *rad52-L264P* cells in comparison to WT cells (Figure 4B and E). Moreover, DSB repair analysis by Southern blotting showed that the kinetics of repair in both systems were unaffected by *rad52-L264P* (Figure 4A and D).

We conclude that the *rad52-L264P* mutation only bypasses *srs2*Δ defects associated with presynaptic Rad51 nucleoprotein filament formation. This finding implies that *rad52-L264P* cannot suppress the high level of CO observed in *srs2*Δ cells either [29], [30]. Southern blot quantification (Figure 4D) confirmed the increased amount of CO in *srs2*Δ cells (18%) compared with WT cells (5%). As expected, the amount of CO in *rad52-L264P srs2*Δ cells was still very high (13%). To confirm this result, we used the *arg4* ectopic recombination system described by Robert et al. [30]. We found CO in 64% of ARG^+^ recombinants in *rad52-L264P srs2*Δ cells in this background, a value comparable to the 60% measured in *srs2*Δ cells (Escartin F, De Cian A, Coïc E, Gilquin B, Le Cam E, Veaute X, unpublished data). Altogether, these results show that the genetic interactions between *srs2*Δ and *rad52-L264P* are related to Rad51 filament formation and not to postsynaptic resolution of intertwined recombination intermediates.

### Persistence of DNA repair checkpoint induced by DSB formation in *srs2*Δ cells is suppressed by *rad52-L264P*

Elimination of the checkpoint response in the *mec1*Δ *sml1*Δ derivative of the *srs2*Δ strain suppresses, like *rad52-L264P*, the poor viability related to the formation of the DSB in the SSA system [28]. As a consequence, we wondered if the Rad52-L264P mutant protein could somehow lower the persistence of the checkpoint related to the maintenance of extensive ssDNA formed around the DSB before repair could take place. The analysis of Rad53 by western blot (Figure 4C) shows that it is similarly phosphorylated two hours after HO induction in both *rad52-L264P* and WT strains. We confirmed that this modification disappears after 12 hours in WT and *rad52-L264P* cells and persists in *srs2*Δ cells even 24 hours after DSB formation. It was previously shown that the persistence of the checkpoint activation in *srs2*Δ cells is dependent on Rad51 [28] because the nucleofilament protects ssDNA from degradation [41]. *rad52-L264P* suppresses the checkpoint activation in *srs2*Δ cells as shown by the similarity of the Rad53 phosphorylation kinetics in *rad52-L264P srs2*Δ compared with WT. This implies that the Rad51 filaments formed by the mutated Rad52 protein allow the checkpoint to be turned off, even in the absence of Srs2. This result strengthens our conclusion concerning the involvement of *rad52-L264P* in the suppression of *srs2*Δ defects in presynaptic Rad51 filament management and suggests that Rad52-L264P-mediated Rad51 filaments are different from Rad52-mediated filaments.

During ectopic gene conversion, the *srs2*Δ mutant defect is associated with a slower disappearance of Rad53 phosphorylation. The retarded bands decrease in intensity after 8 hours in WT cells compared with 12 hours in *srs2*Δ cells (Figure 4F). This slower recovery is not suppressed by *rad52-L264P*, confirming the absence of suppression of *srs2*Δ defects in this system.

### *rad52-L264P* suppresses *srs2*Δ low cell viability in the SSA system through subtle changes in Rad52 mediator activity

**Figure 5:**
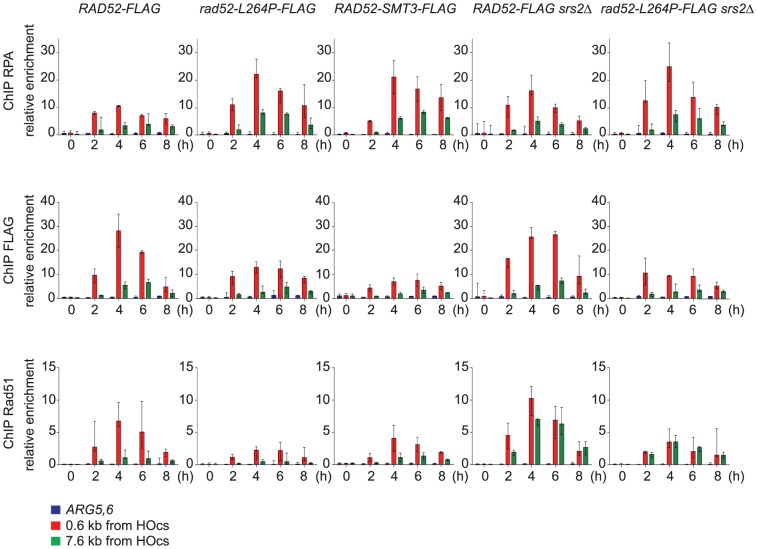
ChIP analysis of Rad51 filament formation at a DSB created by the HO endonuclease in cells that express Rad52-FLAG, Rad52-L264P-FLAG or Rad52-SUMO-FLAG. The HO endonuclease was induced in WT or *srs2*Δ cells that express Rad52-FLAG, Rad52-L264P-FLAG or Rad52-SUMO-FLAG to create a single DSB that can be repaired by SSA. Samples were taken before induction and at 2, 4, 6 and 8 hours after galactose addition. Antibodies against the RPA complex, the FLAG epitope or Rad51 were used to precipitate protein-bound chromatin. Quantitative PCR was performed with primers located at 0.6 kb or 7.6 kb from the DSB site, using the immunoprecipitated chromatin (IP) and input DNA as template. As a control, primers specific for the *ARG5,6* locus were used. The relative enrichment represents the ratio of the PCR enrichment in the IP fraction to the input fraction. The median value of at least 3 experiments is shown and error bars represent the upper and lower values observed.

We carried out chromatin immunoprecipitation (ChIP) experiments to study the recruitment of RPA, Rad52 and Rad51 to ssDNA in order to monitor Rad51 filament formation in cells that express Rad52-FLAG or Rad52-L264P-FLAG. We used the SSA assay described above because DSB repair requires the formation of long ssDNA tails [28], [29]. This allowed us to follow Rad51 filament formation on ssDNA for a long period of time. Quantitative PCR was carried out using primer sets that amplify DNA at 0.6 kb and 7.6 kb upstream of the DSB site during a time-course experiment to follow DSB induction. We found an increase in the relative enrichment of RPA, Rad52-FLAG and Rad51 at the site of DSB formation compared to the uncut control *ARG5,6* locus, indicative of the formation of ssDNA and subsequently of Rad51 filaments (Figure 5). The increase of RPA binding was higher in Rad52-L264P-FLAG than in Rad52-FLAG expressing cells at positions 0.6 kb and 7.6 kb upstream of the DSB site. The highest RPA increase in Rad52-L264P-FLAG expressing cells (2-fold in comparison to what is detected in Rad52-FLAG expressing cells) was observed four hours after HO induction and was associated with a decrease in Rad52-L264P-FLAG and Rad51 binding at the same time point (2- and 3-fold, respectively, in comparison to Rad52-expressing cells, Figure 5). However, Rad51 binding was still 30-fold higher than the enrichment value observed at the *ARG5,6* locus without DSB. The lower Rad51 recruitment in Rad52-L264P-FLAG expressing cells might be caused by a reduced mediator activity of this Rad52 mutant. Alternatively, it might be the result of a modification of the Rad51 filament properties. For example, their average length could be shorter in Rad52-L264P-FLAG than in Rad52-FLAG expressing cells.

### Srs2 removes Rad51 filaments differently according to the distance from the 3′-end of ssDNA tails

We also observed that the recruitment of RPA, Rad52 and Rad51 was lower at position 7.6 kb than at position 0.6 kb in Rad52-FLAG expressing cells (Figure 5). This could be linked to a limited amount of proteins to cover long ssDNA tails. However, western blot analysis of the cell extracts used for ChIP showed that the protein level of RPA and Rad52-FLAG remained unchanged after DSB formation (Figure S3), whereas the pool of Rad51 increased. Consequently, the lower recruitment of Rad51 at 7.6 kb could be related to a specific activity of Srs2 at this position. Indeed, we found that Rad51 recruitment is of the same order of magnitude at the 7.6 kb and 0.6 kb positions in *srs2*Δ cells. The finding that Rad51 binding at 0.6 kb was not significantly affected in *srs2*Δ cells in comparison to WT cells indicates that Srs2 does not influence significantly Rad51 recruitment at position 0.6 kb, while it is very active in eliminating Rad51 filaments at position 7.6 kb. This observation suggests that Rad51 filaments forming far away from the DSB site might be responsible for the death of *srs2*Δ cells upon DSB formation. Finally, reduced Rad51 binding at 7.6 kb was observed also in *rad52-L264P* cells and was confirmed in *rad52-L264P srs2*Δ cells as well. This lower recruitment (or the modification of Rad51 filaments properties) can explain the resistance of such cells to DNA damage.

### *rad52-L264P* does not affect the interaction with RPA, Rad51 and Rad59

**Figure 6:**
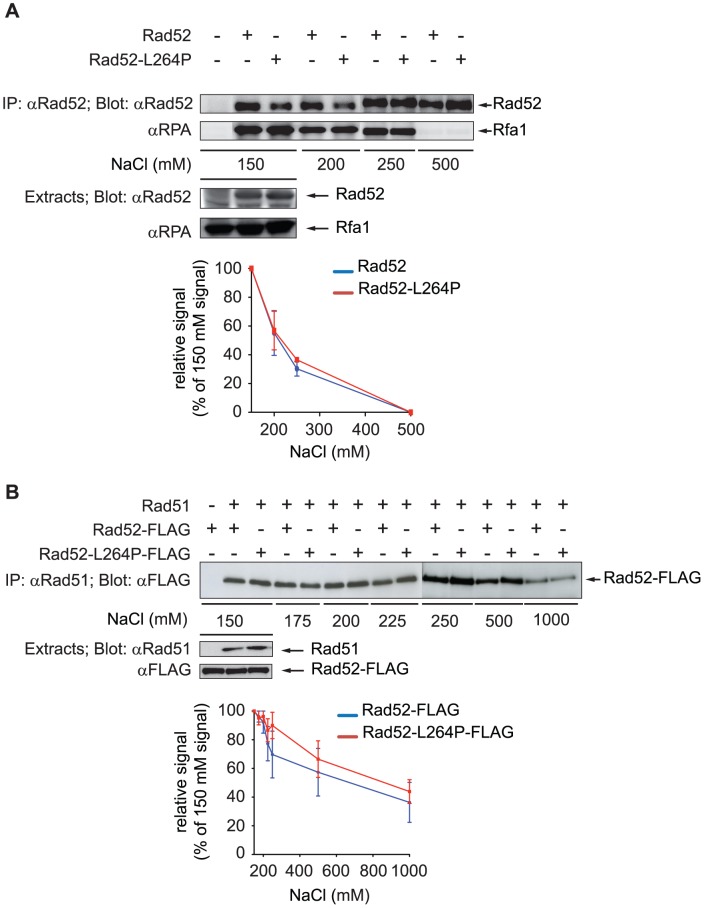
Rad52-L264P still interacts with RPA and Rad51. (A) To test the interaction with RPA, Rad52 or Rad52-L264P was immunoprecipitated with a rabbit anti-Rad52 polyclonal antibody from 1 mg of whole cell extracts (without DNAse treatment) prepared from *RAD52*, *rad52-L264P* or *rad52*Δ strains. To test the robustness of the interaction, increasing NaCl concentrations were added to the cell extracts. Proteins from the whole extracts (50 µg) and from the immunoprecipitated fractions were separated by SDS-PAGE and immunoblotted with rabbit anti-Rad52 polyclonal antibody or rabbit anti-RPA polyclonal antibody (allowing detection of the Rfa1 subunit of RPA). The signals corresponding to immunoprecipitated Rad52 or Rad52-L264P were quantified in three independent experiments and plotted as a fraction of the signal intensity measured in the 150 mM NaCl experiment. (B) To assess the interaction between Rad52-L264P and Rad51, Rad51 was immunoprecipitated from 1 mg of whole cell extracts (without DNAse treatment) from cells expressing Rad52-FLAG or Rad52-L264P-FLAG. The strength of the interaction was also evaluated against increasing NaCl concentrations. The proteins from whole cell extracts (50 µg) and from immunoprecipitated fractions were separated by SDS-PAGE and immunoblotted with rabbit anti-Rad51 polyclonal antibody and mouse anti-FLAG monoclonal antibody. The presence of Rad51 in the immunoprecipitated fraction cannot be detected to validate the efficiency of the immunoprecipitation because it migrates at the same level as the IgG anti-Rad51 used for the immunoprecipitation. However, the absence of Rad52 in the *rad51*Δ immunoprecipitate confirmed that the Rad52-FLAG signals observed are related to the Rad52-Rad51 interaction. The signals corresponding to immunoprecipitated Rad52 or Rad52-L264P were quantified in three independent experiments and plotted as in (A).

We then asked whether the lower recruitment of Rad52-L264P and Rad51 in *rad52-L264P* cells observed by ChIP could be related to a modification of the interaction of RPA or Rad51 with Rad52-L264P. Indeed, L264 is located just upstream of the QDDD motif essential for RPA binding [36] (Figure 1B). Therefore, it was possible that the *rad52-L264P* mutation affects RPA binding [42], [43]. We evaluated Rad52-RPA interaction by co-immunoprecipitation experiments. Rad52 was immunoprecipitated with a polyclonal antibody and the precipitated fraction was analyzed on western blots with a polyclonal antibody directed against the RPA complex (Figure 6A). We first observed that the *rad52-L264P* mutation did not substantially affect the binding to RPA, which was expected for a mutant protein still able to manage DNA repair by HR. However, to make sure that there was no difference between the WT and the mutant protein in binding to RPA, we added increasing salt concentrations to the cell extracts to test the robustness of this interaction. As shown in Figure 6A, the amount of RPA co-immunoprecipitated was equivalent in extracts expressing the WT or the mutant protein. In both cases, the interaction was abolished at 500 mM NaCl. In the same experiment, we looked at the interaction between the mutated Rad52-L264P protein and Rad59 [44] and found no major differences between the WT and the mutant protein at 150 mM NaCl (Figure S2). Since Rad59 interaction is not crucial for Rad52/Rad51-dependent recombination [45], we did not investigate further the effect of *rad52-L264P* on this interaction. We also measured the effect of *rad52-L264P* on Rad51 binding [44]. For that purpose, we used Rad52-FLAG and Rad52-L264P-FLAG tagged proteins and found that the mutation did not affect this interaction (Figure 6B). Similarly to what is observed for RPA, the destabilization of the interaction with Rad51 by increasing NaCl concentrations was comparable when using WT Rad52 or Rad52-L264P.

### Purified Rad52-L264P is more efficient in Rad51 nucleoprotein filament formation than the WT protein

**Figure 7:**
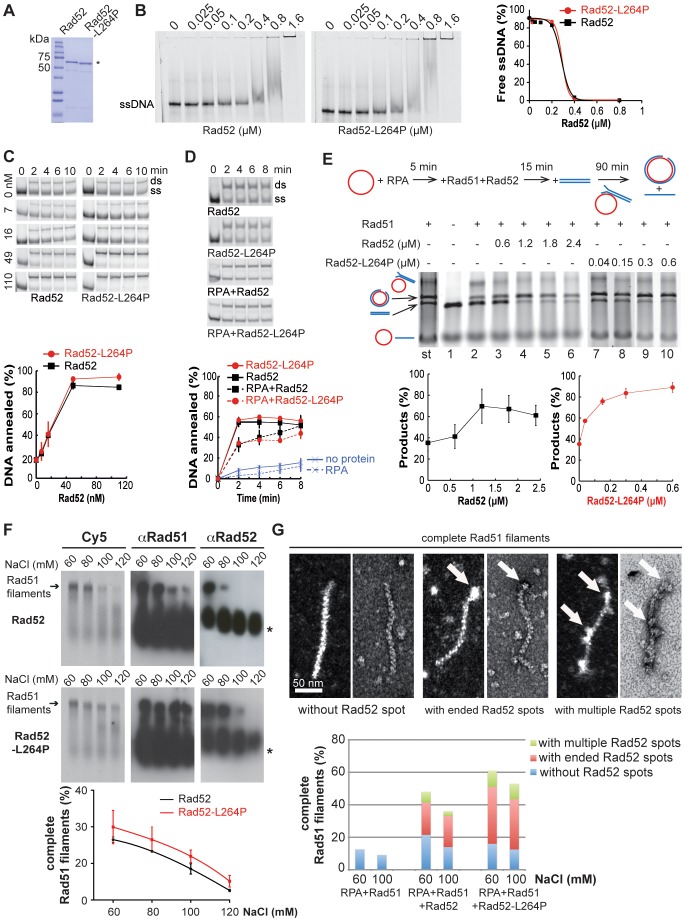
Biochemical analysis of Rad52-L264P-mediated filaments. (**A**) The purity of recombinant Rad52 and Rad52-L264P (2 µg/each) was assessed by separation on 8% SDS-PAGE and staining with Coomassie blue. (B) Binding of Rad52-L264P to ssDNA. Protein titration reactions were performed by incubating 0.27 µM of a 62-nucleotides long Cy5-labeled ssDNA fragment with various amounts of Rad52-L264P at 37°C for 10 min (protein/bases: 1/10, 1/5, 1/2.5, 1/1.25, 1/0.6, 1/0.3, 1/0.15). Quantification of free ssDNA is shown. The data were fitted into a sigmoidal curve by using the PRISM software (GraphPad). (C) Rad52-L264P-mediated DNA annealing. Representative gels of Rad52 or Rad52-L264P-promoted DNA annealing are shown in the upper panel (Rad52/bases: 1/100, 1/42, 1/14, 1/6; same DNA as in (B) with reverse-complement, 340 nM each). The dsDNA/total DNA ratio at 10 min is shown in the lower panel. (D) RPA bound to ssDNA inhibits equally Rad52- and Rad52-L264P-catalyzed annealing reactions. Reactions were carried out with primers 25 and 26 (200 nM each, see Material and Methods) that were first incubated with 30 nM RPA (1/13 bases) at 30°C for 5 minutes, followed by addition of 40 nM Rad52 (1/10 bases). Self-annealing of the primers incubated without proteins and reactions performed without RPA or Rad52 are also shown. (E) Over-stimulation of DNA strand exchange by Rad52-L264P. *Upper panel*, diagram of the reaction substrates and products. *Middle panel*, ethidium bromide-stained DNA gel. As shown by the standard reaction (st), Rad51 efficiently catalyzes the formation of nicked circular products. Pre-bound RPA inhibits this reaction (line 2). Increasing amounts of Rad52 (lines 3–6, Rad52/bases: 1/55, 1/27, 1/18, 1/14) or Rad52-L264P (lines 7–10, Rad52/bases: 1/880, 1/220, 1/110, 1/55) overcome the inhibitory effect of pre-bound RPA. In line 1, only RPA was added to the reaction. *Lower panel*, the ratio of the nicked circular product over the sum of the linear dsDNA substrate and the nicked circular product is shown. (F) Salt titration of Rad51-Rad52/Rad52-L264P-ssDNA complex formation. The nucleoprotein complexes were assembled by incubating 0.8 µM Rad51 with 0.09 µM Rad52 or Rad52-L264P and 0.08 µM RPA pre-bound to 2.5 µM Cy5-labeled ssDNA (400 nucleotides) in the presence of the indicated NaCl concentrations at 37°C for 15 min. The Cy5 signals after nucleoprotein gel eletrophoresis are shown. Quantifications are shown below. Data were fitted into a third order polynomial curve. Western blot analysis of the gel using antibodies against Rad51 or Rad52 is also shown. Stars indicate signals corresponding to proteins not bound to ssDNA. (G) Transmission electron microscopy images of protein-DNA complexes showing the association of Rad52 with complete Rad51 filaments. Positive (left) and negative (right) staining images are shown for each type of filaments. The proportion of each type of complete Rad51 filaments formed by Rad52 or Rad52-L264P at different NaCl concentrations is shown and compared to a control reaction without Rad52. 100 molecules were examined in each experiment.

To characterize the biochemical properties of Rad52-L264P we purified recombinant Rad52-L264P and Rad52 from *Escherichia coli* (Figure 7A). Using electrophoretic mobility shift assays (Figure 7B), we found that Rad52 binding to ssDNA was not significantly affected by the mutation. Next, we investigated whether the L264P mutation affected Rad52 annealing activity by incubating Rad52-L264P and WT Rad52 with complementary ssDNA strands and monitoring the formation of dsDNA. Again, no significant difference was observed (Figure 7C). This was further confirmed in a reaction where ssDNA was coated first with RPA to reflect the *in vivo* conditions (Figure 7D). We also observed that Rad52-L264P annealing activity was sensitive to RPA, as previously reported for WT Rad52 [7]. Finally, Rad52-L264P and Rad52 annealing activity were similarly affected by Rad51 filaments and free Rad51 proteins [7] (Figure S4A–C).

To determine whether the L264P mutation has an impact on Rad52 recombination mediator activity, we used a well-established DNA strand exchange system that involves plasmid DNA substrates (Figure 7E). In this system, RPA that is pre-bound to ssDNA partially inhibits strand exchange by Rad51. The addition of Rad52 together with Rad51 increases the formation of products through Rad52 interaction with RPA (reviewed in [4]). We found that Rad52-L264P was approximately 8-fold more efficient than WT Rad52 in catalyzing DNA strand exchange.

As this finding indicates that the mediator activity of Rad52-L264P is modified, we studied more precisely the effect of the L264P mutation on the formation of Rad51 filaments and their stability by using electrophoretic analysis of glutaraldehyde-fixed Cy5-labeled DNA-protein complexes. First, we optimized RPA, Rad51 and Rad52 stoichiometry at 60 mM NaCl to obtain the best Rad52 antagonism of the inhibitory effect of pre-bound RPA on Rad51 nucleoprotein filament formation (Figure S5). In these conditions, Rad52-L264P showed a slightly increased mediator activity in comparison to WT Rad52 (Figure 7F). Challenging Rad51 filament formation with increasing salt concentrations showed that the mediator activity of both WT and mutant Rad52 was very sensitive to salt. Indeed, the addition of only 60 mM NaCl to the reaction (120 mM in total) was sufficient to reduce Rad51 filament formation from 30 to 10% of the protein/DNA complexes. Quantification of Rad51 filament formation at increasing NaCl concentrations confirmed that Rad52-L264P was slightly more efficient than WT Rad52 (the difference was significant at 100 and 120 mM NaCl). However, the salt titration midpoint was the same for both WT and mutant protein (around 90 mM), again indicating that the difference in the mediator activity between Rad52-L264P and Rad52 is minimal. Western blot analysis using an anti-Rad51 polyclonal antibody confirmed the slightly higher mediator activity of Rad52-L264P. Electron microscopy (EM) analysis of the resulting protein-ssDNA complexes confirmed the sensitivity of the reaction to NaCl concentration and the higher mediator activity of Rad52-L264P (Figure 7G). We also measured the stability of the nucleoprotein filaments formed by Rad52 or Rad52-L264P by adding increasing concentrations of NaCl after Rad51 filament formation (Figure S5B) and found no difference. The salt-titration mid-point was around 400 mM.

In addition, western blot analysis using an anti-Rad52 polyclonal antibody revealed that Rad52 remained associated with complete Rad51 filaments (Figures 7F and S5). This association was quite unstable because the addition of 60 mM NaCl after complete formation of filaments resulted in Rad52 dissociation from the complex (Figure S5C). EM analysis of the nucleoprotein complexes formed with Rad52 (at 60 mM NaCl) showed that 55% of complete Rad51 filaments remained associated with the mediator protein (Figures 7F and S5). Rad52 was localized mostly at the end of filaments (75%), but in many cases multiple Rad52 spots were distributed all along the filament. In absence of RPA pre-bound to ssDNA, Rad52 was rarely associated with Rad51 filaments (Figure S5A). Thus, Rad52 spot formation might be dependent on the previous binding of RPA to ssDNA. Rad52 might remain associated with residual RPA bound to DNA between Rad51 molecules. Alternatively, as only a few complete Rad51 filaments are formed in the absence of RPA, Rad52 association might be restricted to complete Rad51 filaments. Our results also show that Rad52-L264P association with Rad51 filaments was increased (74% of complete Rad51 filaments compared with 55% for WT Rad52, Figure 7G and S5), while the proportion of Rad52-L264P spots at the end of filaments was comparable (78%). This increased association of Rad52-L264P together with its higher mediator activity might modify qualitatively Rad51 filament properties and cause the suppression of *srs2*Δ phenotypes *in vivo*.

### *SIZ2* overexpression suppresses the MMS sensitivity of *srs2*Δ cells via Rad52 sumoylation

**Figure 8:**
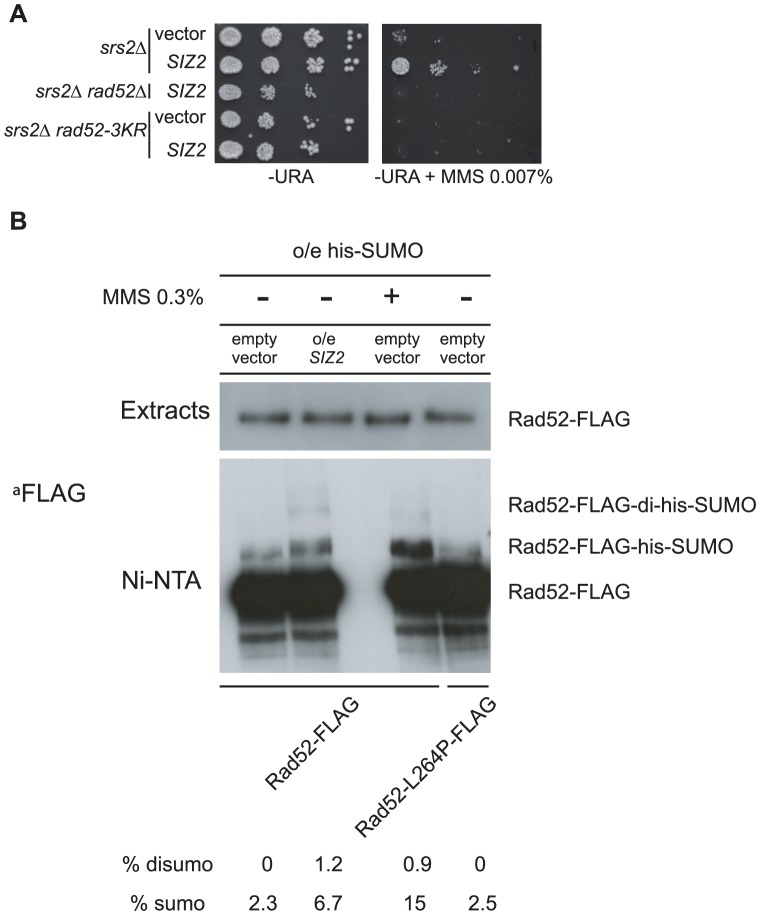
Over-expression of the *SIZ2* SUMO-ligase coding gene suppresses the MMS sensitivity of *srs2*Δ by stimulating Rad52 sumoylation. (A) Spot assay of haploid cells over-expressing *SIZ2*. Serial 10-fold dilutions were plated on minimal media lacking uracil with or without MMS. Strains of the indicated genotype were transformed with an empty vector or with the same plasmid containing the *SIZ2* gene. (B) Over-expression of *SIZ2* stimulates Rad52 sumoylation. Proteins conjugated with a His_7_-SUMO radical were pull-down on Ni-NTA from 5 mg of extracts of *RAD52*-*FLAG* cells over-expressing His7-*SMT3*. Pull-downs were carried out from strains transformed with a *SIZ2*-containing multi-copy vector or with an empty vector. Cells treated with 0.3% of MMS were also tested as a positive control of sumoylation. *rad52-L264P-FLAG* strains were also subjected to pull-down analysis. Proteins from the whole extracts (3 µg) and from the pull-down fractions were separated by SDS-PAGE and immunoblotted with an anti-FLAG mouse monoclonal antibody.

In parallel to the search for point mutations suppressing the MMS sensitivity of *srs2*Δ mutants, we also screened a multi-copy genomic library for high-dosage suppressors. After a second round of selection, plasmids bearing a suppressor were sequenced. Strikingly, we isolated a plasmid carrying the *SIZ2* gene (Figure 8A). Siz2 is the only SUMO ligase involved in Rad52 sumoylation after the formation of chemically induced DNA damage (like MMS) [11]. Consequently, we speculated that the stimulation of Rad52 sumoylation was responsible for the suppression conferred by *SIZ2* overexpression since our study of *rad52-L264P* shows that subtle changes in Rad52 activity can bypass the requirement for Srs2 in haploid cells. Indeed, we found that the suppression of the MMS sensitivity of *srs2*Δ conferred by *SIZ2* overexpression is no longer observed in cells bearing a sumoylation-deficient *rad52-3KR* allele, where the three SUMO acceptor sites, lysines 10, 11 and 220 are replaced by arginines [11] (Figure 8A). As a control, we checked that the *rad52-3KR* allele was not sensitive to MMS (Figure 9B and D), as previously described [11]. Therefore, the suppression of the MMS sensitivity of *srs2*Δ cells by the overexpression of *SIZ2* occurs through the sumoylation of Rad52.

To check that *SIZ2* overexpression truly stimulates Rad52 sumoylation, extracts from cells overexpressing *SIZ2* and His-tagged SUMO were subjected to Ni-NTA pull-down experiments under denaturing conditions (Figure 8B). The amount of Rad52 sumoylation was compared to that of cells expressing *SIZ2* under normal physiological conditions. Because the FLAG tag we used to detect Rad52 contains a poly-histidine chain, the protein was also retained on the Ni-NTA beads, allowing us to quantify the amount of sumoylated protein relative to the total amount of Rad52. Overexpression of *SIZ2* increases the amount of sumoylated Rad52 3-fold compared with its basal expression. This result strengthens our genetic experiments establishing that an increase in the pool of sumoylated Rad52 allows Srs2 activity to be bypassed.

### Rad52-L264P behaves as Rad52-SUMO

Since Rad52 sumoylation leads to the suppression of MMS sensitivity of *srs2*Δ cells, we wondered whether the suppression by *rad52-L264P* was dependent on an increase in the pool of sumoylated Rad52 induced by the mutation. As shown in Figure 8B, *rad52-L264P* does not increase Rad52 sumoylation. We also found that the suppression conferred by *rad52-L264P* was not dependent on *SIZ2* since the deletion of this gene in *rad52-L264P srs2*Δ cells did not affect MMS resistance (Figure 9A). Additionally, a *rad52-L264P* allele that cannot be sumoylated (*rad52-3KR-L264P*) still suppresses the MMS sensitivity of *srs2*Δ mutants (Figure 9B). Altogether, these results clearly show that Siz2-mediated Rad52-L264P sumoylation is not required to bypass Srs2. We also checked if the Rad52-L264P protein was sumoylated by another SUMO ligase. We found that Rad52-L264P MMS-induced sumoylation was still dependent on Siz2 (Figure S6). To reconcile our findings that *srs2*Δ MMS sensitivity can be suppressed on the one hand by Rad52 sumoylation and on the other hand by *rad52-L264P*, even if sumoylation cannot occur, we propose that Rad52-L264P behaves as if it is constitutively sumoylated, even when it is not physically modified. This would mean that the mutation has the same effects on Rad52 activities, as does sumoylation. Consequently, cells that constitutively express only sumoylated Rad52 should suppress the MMS sensitivity of *srs2*Δ cells as strongly as *rad52-L264P*.

To test this hypothesis, we fused the *SMT3* gene, coding for the SUMO modifier, to the 3′ end of the endogenous *RAD52* gene, to generate cells expressing only a Rad52 protein bearing a C-terminal SUMO fusion. The part of *SMT3* coding for the last three amino acids and the two glycines required for conjugation [46] was removed to avoid subsequent conjugation of SUMO with other proteins. The resulting strain displays only mild MMS sensitivity (Figure 9C), showing that the addition of SUMO does not substantially affect Rad52 activity in DNA repair. *srs2*Δ cells bearing the *RAD52-SMT3* fusion allele were as MMS-resistant as those carrying *rad52-L264P*. Therefore, in cells that produce only Rad52-SUMO fusion proteins, Srs2 becomes incidental to MMS resistance. We also monitored the effect of the fusion protein on survival of *srs2*Δ cells in both SSA and *MAT* ectopic recombination systems (Figure 4B and E). As for Rad52-L264P, the expression of the Rad52-SUMO protein instead of Rad52 can alleviate the strong lethality associated with *srs2*Δ in the SSA system but not in the ectopic system. Additionally, ChIP analysis showed that Rad52-SUMO behaves as Rad52-L264P. Using FLAG-tagged proteins, we also observed a 2-fold decrease in Rad52-SUMO recruitment to ssDNA compared with Rad52 (Figure 5). This is associated with a 2-fold decrease in Rad51 binding, as we observed for Rad52-L264P. These results reinforce the idea that Rad52 sumoylation affects Rad52 activities in the same way as *rad52-L264P*.

Since Rad52 sumoylation can bypass the requirement for Srs2 in the avoidance of toxic Rad51 filaments, it would be expected that the expression of the Rad52-3KR protein, which cannot be sumoylated, would lead to a negative interaction with *srs2*Δ. This is exactly what we observed (Figure 9D). This negative effect is also observed in a *siz2*Δ *srs2*Δ double mutant (Figure 9E). It is interesting to note that the negative effect of *rad52-3KR* in a *srs2*Δ background is dominant to the *RAD52* allele. Indeed, the introduction of the *RAD52* allele in a *rad52-3KR srs2*Δ strain does not increase MMS resistance (Figure 9D). We suggest that Rad52-3KR mediates toxic Rad51 filaments that can be disassembled only by Srs2.

It has been reported that a plasmid carrying *rad52-3KR* is able to suppress (albeit weakly) *rrm3Δ srs2*Δ synthetic lethality and *sgs1*Δ *srs2*Δ growth defect [11]. However, we found that *rad52-L264P* suppresses these negative interactions (Figure 3A), while this allele codes for a protein behaving as if it were sumoylated. We confirmed our conclusion by tetrad analysis showing that a single integrated copy of *rad52-3KR*, or *siz2*Δ, cannot rescue *rrm3*Δ *srs2*Δ and *sgs1*Δ *srs2*Δ negative interactions (Figure 3B).

Finally, it has been reported that the Rad52-3KR protein is more prone to proteasome degradation than the WT protein, mostly in cells lacking Srs2 or Rrm3 helicases [11]. Since *rad52-L264P* makes the protein behave as if it were constitutively sumoylated, it was possible that it could suppress the increased instability of Rad52-3KR. Expression shut-off experiments were performed with different mutation of Rad52 in *srs2*Δ mutants. We found that the Rad52-3KR-L264P protein was not more stable than Rad52-3KR (Figure S7). It is possible that the decreased stability of Rad52-3KR is related to the substitution of the three targeted lysines and not to the inability of the protein to be sumoylated.

## Discussion

### A novel allele of *RAD52* circumvents the requirement for Srs2 activity in DNA repair

We found a novel allele, *rad52-L264P*, which suppresses the requirement for Srs2 activity in DNA damage tolerance. A profusion of *RAD52* mutants displaying a separation of function has been studied, revealing the multifunctional nature of the protein. Some of them were affected in DSB repair activity but not in spontaneous recombination [34], [47]. Others were differentially affected in the mediator and the ssDNA annealing activity of Rad52 [48]–[50]. Some of these mutants, which were shown to affect Rad52 mediator activity, display a partial resistance to DNA damage [49], [50], but their viability is still reduced by several orders of magnitude. Lastly, some have been shown to alter the choice of the donor template during spontaneous HR [51]. *rad52-L264P* contrasts with all these mutations because it does not confer any of the *rad52*Δ null mutation-associated phenotypes (defect in vegetative growth, increased spontaneous mutagenesis, very high MMS and γ-ray sensitivities, large decrease in DSB repair by SSA and gene conversion). In addition, it is not a hypomorphic allele since it is co-dominant when it is combined with the WT allele.

Several alleles of *RAD52* that partially suppressed the sensitivity of *srs2*Δ cells to DNA damage have been previously described [20]. They were all highly defective in DNA repair and HR, but not as much as the null allele. The viability of cells carrying these alleles is reduced 10- to 20-fold at low doses of MMS, and HR is reduced 10-fold. They were all dominant-negative and, unlike what we observed for *rad52-L264P*, they have to be combined with the WT allele or overexpressed to suppress only partially the MMS or UV sensitivities of *srs2*Δ mutants. In addition, none of these *RAD52* mutants suppressed both phenotypes, while this is the case for *rad52-L264P*. It was proposed that these *rad52* alleles are able to rescue *srs2*Δ mutants partially by reducing HR efficiency. Some of these mutations code for C-terminal truncations unable to bind Rad51. Therefore, they could bind ssDNA without forming Rad51 filaments. Others were N-terminal truncations, probably impaired in DNA binding, which suggests that their overexpression resulted in depletion of the protomer pool of Rad51. It has been proposed that both kinds of mutations result in a large decrease in Rad51 filament formation, resulting in a limited suppression of *srs2*Δ MMS or UV sensitivities. However, *rad52-L264P* suppresses many defects of *srs2*Δ haploid cells without displaying any *rad52*Δ phenotype. Thus, in contrast with previously described alleles, *rad52-L264P* allows an extensive genetic and molecular investigation of the toxic recombination intermediates formed by Rad52 and eliminated by Srs2 in WT cells.

### *rad52-L264P* specifically bypasses the role of Srs2 in the removal of toxic Rad51 filaments

**Figure 10:**
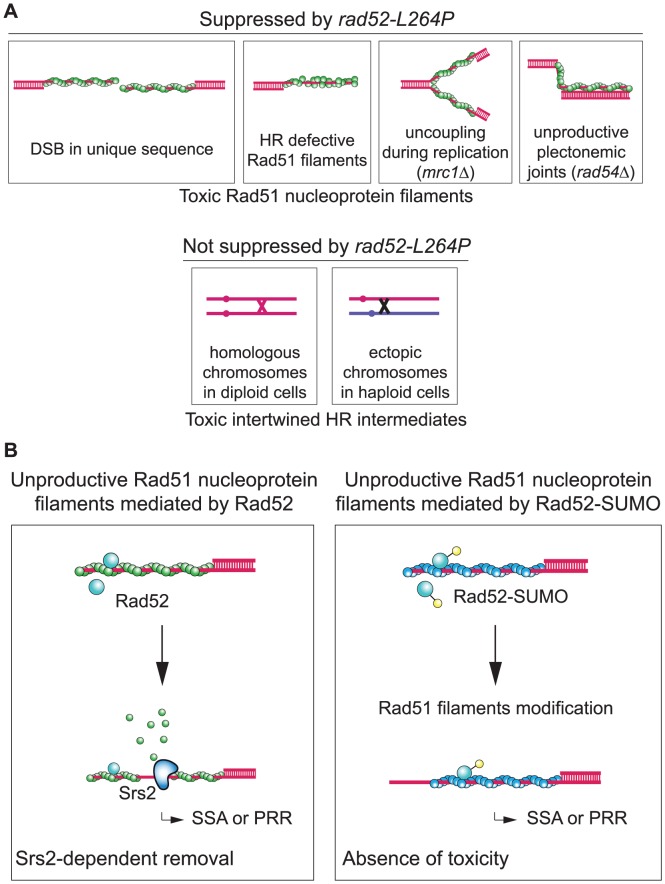
Rad52 sumoylation prevents the toxicity of unproductive Rad51 filaments. (A) Schematic representation of the two kinds of toxic recombination intermediates eliminated by Srs2 in WT cells. We found that *srs2*Δ haploid cells sensitivity to DNA damage is related to recombination-deficient Rad51 nucleoprotein filaments. The toxicity of such filaments disappears in *rad52-L264P* srs2Δ cells, or alternatively, the intermediates themselves are not formed. However, *srs2*Δ cells sensitivity to DNA damage related with toxic intertwined HR intermediates cannot be suppressed by this allele. Unproductive Rad51 filaments can be formed after resection of a DSB located in a unique sequence in the genome. In a situation where homologous dsDNA cannot be found by the recombinase, Srs2 is essential to remove Rad51 filaments to allow alternative repair pathways such as SSA. Srs2 could also edit Rad51 filaments improperly nucleated by Rad52. In *srs2*Δ cells, nonrecombinogenic Rad51 filaments could also accumulate on ssDNAs generated from the uncoupling between the helicase complex opening replicative dsDNA and the DNA synthesis machinery in replicative mutants such as *mrc1*Δ. Finally, when a stable paranemic joint cannot be processed from a plectonemic joint because of mutations in genes involved in late recombination steps, Srs2 is necessary to address lesions to other DNA repair pathways. Intertwined recombination intermediates that occur between homologous chromosomes in diploid cells or between ectopic chromosomes in haploid cells cannot be suppressed by *rad52-L264P*. (B) Unproductive Rad51 filaments mediated by Rad52-SUMO are not toxic even in *srs2*Δ cells. When mediated by Rad52, Rad51 filaments that cannot complete strand invasion have to be removed by Srs2 in order to allow SSA or post-replication repair processes (PRR). Conversely, Rad52-SUMO (or Rad52-L264P) might lower (or shorten) Rad51 filaments. These modified mediators might also change Rad51 filament properties as indicated by Rad52 occupancy on Rad51 filaments. These changes might suppress Rad51 filaments toxicity, thereby bypassing the need for Srs2. Rad51 filaments might be removed by a Srs2-independent process.

We found that *rad52-L264P* cannot overcome the function of Srs2 in the resolution of postsynaptic recombination intermediates, but only those associated with the removal of presynaptic Rad51 filaments. It was reported previously that the Srs2 anti-recombination function in removing toxic Rad51 filaments is genetically separable from its role in promoting the resolution of postsynaptic recombination intermediates, which depends exclusively on Srs2 Cdk1-dependent phosphorylation [52]. Our results are in agreement with this finding. *rad52-L264P* does not suppress the increased sensitivity to UV and γ-ray irradiation of *srs2*Δ/*srs2*Δ diploid cells. It is also unable to suppress the strong lethality associated with ectopic HR in *srs2*Δ cells. Additionally, it cannot suppress the high rate of CO found in these cells. We propose that these defects are related to the resolution of HR intermediates between homologous or ectopic chromosomes that absolutely requires the helicase activity of Srs2 (Figure 10A). Additionally, our results support the recent finding that the post-synaptic role of Srs2 is to dismantle HJs through its helicase activity [32] rather than by displacing Rad51. However, *rad52-L264P* perfectly rescues the lethality associated with DSB repair by SSA between distant repeats. In this assay, a Rad51-dependent strand invasion step is not involved in repair [28]. Therefore, Srs2 is required to remove Rad51 filaments forming on ssDNA around the initial DSB to allow proper repair. By extension, our findings suggest that Rad51 filaments are also responsible for *srs2*Δ defects after γ-ray and UV irradiation and in cells bearing mutations in genes that impair replication or recombination (Figure 10A).

### The nature of toxic Rad51 filaments eliminated by Srs2

In all the situations where the requirement for Srs2 can be bypassed by *rad52-L264P*, we propose that the formation of extensive ssDNA may potentially lead to the establishment of Rad51 filaments that cannot recombine (Figure 10A). These “unproductive” filaments would be a signal to trigger Srs2 translocase activity in WT cells. In the SSA assay, ChIP experiments showed that Srs2 is more active in removing Rad51 at 7.6 kb than at 0.6 kb from the DSB site, thus indicating how Srs2 might interfere with such unproductive filaments in WT cells. Indeed, Srs2 job might be to remove extended rather than short Rad51 filaments located near the 3′-end of the ssDNA tail. The length of 5′-ssDNA resection is in part related to strand invasion. Indeed, it is well documented that the formation of a DSB in a unique sequence results in extended resection [53]. Therefore, the formation of extended Rad51 filaments, which is a potential mark of strand invasion failure, would trigger Srs2 activity. The formation of filaments near the ssDNA end might be strictly controlled, while the lower recruitment of Rad52 at more distant sites might affect the quality of the filaments, triggering Srs2 activity. This would allow DSB repair by SSA to occur when HR is inefficient. In *srs2*Δ cells, the persistence of extended, toxic Rad51 filaments would lead to DSB repair towards a dead-end.

Likewise, synthetic lethality of *srs2*Δ with replication mutants, like *mrc1*Δ, would be related to unproductive Rad51 filament formation. In *mrc1*Δ cells, uncoupling between DNA synthesis and DNA unwinding by the MCM helicases [54] leads to the formation of complementary ssDNAs on each newly replicated sister chromatid. In the absence of Srs2, Rad51 filaments formed on these ssDNAs would not be productive because of the lack of a dsDNA template on each sister chromatid. Such filaments could impede replication fork restart. Considering that Srs2 is more active on Rad51 filaments not associated with a 3′-end, we suggest that Srs2 might specifically recognize and eliminate Rad51 filaments formed on parental ssDNA at the replication fork because they are not associated with such an end.

The precise nature of toxic recombination intermediates in *rad50*Δ, *rad54*Δ or *sgs1*Δ has yet to be clarified. Rad50 has an essential role in sister-chromatid recombination (reviewed in [55]) and Rad54 is necessary to initiate DNA synthesis after the formation of the D-loop [56], to extend this structure [57] and to remove the dsDNA template nucleosomes [58]. Sgs1 is probably involved in both replication and recombination mechanisms. Sgs1 could help replication progression and could prevent the formation of ssDNA on which Rad51 filaments could nucleate in *srs2*Δ cells [40]. Furthermore, several lines of evidence suggest that Sgs1 is also involved in the dissolution of dHJ [59], [60] and it was proposed that in *sgs1*Δ cells Srs2 could prevent the formation of these structures [40]. Therefore, in all these backgrounds, toxic recombination intermediates accumulating in the absence of Srs2 could be presynaptic Rad51 filaments or unprocessed postsynaptic recombination intermediates. However, since *rad52-L264P* can suppress *rad50*Δ, *rad54*Δ and *sgs1*Δ synthetic growth defect or lethality with *srs2*Δ, it seems more relevant that these negative interactions are the consequence of the accumulation of ineffective Rad51 filaments.

Finally, γ-ray and UV irradiation would sensitize *srs2*Δ cells because of the formation of unproductive Rad51 filaments on extensive ssDNA. These would accumulate because of DNA damage-induced fork stalling or unfulfilled DNA repair.

In contrast, *rad52-L264P* cannot suppress the increased sensitivity to UV and γ-ray irradiation of *srs2*Δ/*srs2*Δ diploid cells compared with *srs2*Δ haploids. Nor can it suppress the inability to repair a DSB by ectopic recombination nor the high level of CO associated with gene conversion of *srs2*Δ haploid cells. We propose that these defects are related to the resolution of HR intermediates between homologous or ectopic chromosomes which absolutely requires the helicase activity of Srs2 (Figure 10A).

### How does Rad52-L264P bypass Srs2?

*In vitro* experiments showed that Rad52-L264P mediator and strand exchange activities are stronger than those of WT Rad52. This intriguing gain of function of Rad52-L264P is not correlated with any modification of its binding to RPA or Rad51 *in vivo*. Thus, further biochemical analyses of Rad52-L264P are required to provide valuable insights on the mechanism by which Rad52 mediates Rad51 filament formation. *In vivo*, other Rad51 filament mediators, which might act with Rad52 as a complex, could be affected by the *rad52-L264P* mutation. This would explain the reduction in Rad51 binding associated with increased RPA recruitment, measured by ChIP, in *rad52-L264P* cells, while, *in vitro*, Rad52-L264P is a better mediator than the WT protein. The evidence that Rad52-L264P restricts the formation of (or shortens) Rad51 filaments without affecting HR efficiency could explain how this mutation can bypass Srs2, not only in SSA, but also after irradiation or in mutants that are synthetically lethal with *srs2*Δ.

Alternatively, Rad52-L264P could change substantially the properties of Rad51 filaments. Western blot analysis and EM images of Rad51 filaments nucleated *in vitro* show that Rad52 remains associated with complete Rad51 filaments. This association is weak because Rad52 is released by the addition of only 60 mM NaCl, but it could be stabilized *in vivo* by chaperone proteins. The presence of Rad52 within the Rad51 filament might have consequences on homology search and strand invasion. Indeed, Rad52-L264P association with Rad51 filaments is increased in comparison to WT Rad52, a result that can be correlated with the more efficient strand exchange activity observed with the mutant protein. Rad52-L264P might also affect Rad51 filament properties in a way that would suppress their potential toxicity in *srs2*Δ cells. For example, such filaments would not prevent the restart of stalled replication forks, thereby bypassing the need for Srs2.

### Sumoylation affects Rad52 in the same way as the *rad52-L264P* mutation

We also found that Rad52-SUMO fusion protein and Rad52-L264P show a similar ability to avoid or restrict the formation of toxic Rad51 filaments. It was previously reported that Rad52 is sumoylated simultaneously with RPA and Rad59 following treatment with a high dose of MMS [15]. This sumoylation wave might stabilize complexes engaged on their substrates rather than to promote specificity. However, recent work showed that Cdc48 interacts with sumoylated Rad52 and consequently dissociates it from DNA [16]. This interaction might be part of another Rad51 filament formation regulation process, acting in parallel with Srs2 activity. Here, we demonstrate that increasing the pool of sumoylated Rad52 suppresses *srs2*Δ deficiencies in haploid cells. We thus propose that Rad52 sumoylation might modulate its mediator activity and/or change the properties of the formed Rad51 filaments, maybe through Cdc48 activity, in the same way as Rad52-L264P (Figure 10B).

It seems likely that sumoylation of Rad52 is a conserved process because mono- and disumoylation of human Rad52 were also observed in HEK293T cells [11]. It would be interesting to know if this modification induces changes in Rad51 filament properties in mammals as it does in yeast. In this case, it might be important to explore the genetic interactions between Rad52 sumoylation and the newly characterized anti-recombinase PARI [61], which could be the mammalian Srs2 ortholog. Lastly, it is tempting to compare the potential differences between Rad52 un-sumoylated and sumoylated proteins in yeast and those of Rad52 and BRCA2 in metazoans. Recently, it was shown in human cells that these two proteins co-exist as mediators of the Rad51 filaments [62]. Even if the roles of Rad52 in recombination in yeast and metazoans are certainly different, it is possible that in both cases different Rad51 filaments are nucleated.

## Materials and Methods

### Plasmids

For integration into the yeast genome, the *rad52-L264P* allele was cloned into the Yiplac211 integrative plasmid. *rad52-L264P* was PCR amplified from genomic DNA with primers containing restriction sites suitable for cloning. The digested PCR product was ligated into the *Eco*RI and *Hind*III sites of Yiplac211 to give YipLac211-*rad52-L264P*. YipLac211-*rad52L264A* was made by directed mutagenesis from YipLac211-*rad52-L264P* (Phusion Site-Directed Mutagenesis kit, Finnzymes) with primers changing the CCC codon coding for P264 to a GCC codon coding for A264. The *rad52-L264P* mutation was introduced in the same way into pYI211::Kan-*rad52-K10,11,220R* (D2535, provided by S. Jentsch) with primers changing the CTC codon coding for L264 to a CCC codon coding for P264. Yep181-CUP-His_7_-Smt3 [63] was used to overexpress His_7_-Smt3 in cells in order to immunoprecipitate SUMO-conjugated proteins. To create a fusion between *RAD52* and *SMT3*, the *SMT3* ORF was fused to the 3′ intergenic sequences of *RAD52* in a vector bearing the *NATMX* cassette coding for the resistance to clo-NAT (pAG25, [64]). First, the *SMT3* ORF was PCR amplified from FF18733 genomic DNA with primers suitable for cloning. The last three amino acids and the two glycines required for conjugation [46] were removed to avoid subsequent interaction of SUMO with other proteins. The natural *SMT3* stop codon was conserved. The PCR product was ligated into the *Hind*III and *Bam*HI sites of pAG25 resulting in pAG25-*SMT3*. This construct created a *Nhe*I restriction site just 5 bp before the *Bam*HI sites. The *RAD52* 3′ intergenic sequence was then ligated into the *Nhe*I and *Bam*HI sites of this plasmid, generating pEC54. p*SIZ2* is a subclone of a plasmid suppressing the MMS sensitivity of *srs2*Δ cells isolated from an overexpression library built for this study.

### Yeast strains

Strains used in this study are listed in Table S2. Experiments were mostly conducted in the FF18733 background. Diploid cells used in survival and recombination assays were the result of crosses between two different cell backgrounds: FF18733 and FF18985 in order to monitor HR between the *his7-1* and *his7-2* alleles. All the deletion mutants were constructed by the one-step gene disruption method [65]. Multiple mutant strains were derived from meiotic segregants from FF18733 or FF18985 isogenic diploids. Mutations *rad52-L264P* and *rad52-L264A* were introduced into yeast cells with the pop-in pop-out technique using the integrative plasmids Yiplac211-*rad52-L264P* and Yiplac211-*rad52-L264A*. The non-sumoylable *rad52-3KR* and *rad52-3KR*-*L264P* alleles were targeted at the *URA3* locus by transformation of the integrative plasmids pYI211::Kan-*rad52-K10,11,220R* (D2535 [11]) and pYI211::Kan-*rad52-K10,11,220R-L264P*. The *SMT3* gene, coding for the SUMO radical, was fused *in vivo* to the 3′ end of the *RAD52* gene. The insert of pEC54, containing the *SMT3* ORF and a *NATMX* resistance cassette, was PCR amplified with primers designed to introduce *SMT3* in phase with *RAD52*, without affecting the intergenic sequences surrounding *RAD52*. Transformants were selected on clo-NAT containing medium and checked by colony PCR. The production of the fusion protein was checked on western blot (Figure S8). The strain bearing the Rad59-9xMYC fusion protein was made using the method described in [66] in the FF18733 background. The Rad52-FLAG strains were constructed as previously described in the same background [67].

### Sequence alignment

Homologous sequences of *S. cerevisiae* Rad52 were retrieved using PSI-Blast searches against the nr database [68], [69]. A multiple sequence alignment of the full-length sequences of these homologs was obtained using Muscle software [70]. However, within the C-terminal disordered tail the algorithm did not satisfactorily align the small linear motifs surrounding L264 and the alignment had to be manually refined. Propensities to adopt secondary structures were estimated using PsiPred restricting the alignments to subsets of species such as those from the Hemiascomycetes group [71]. The final alignment was represented using Jalview [72].

### Irradiation and induced recombination

UV irradiation was performed using a 264 nm source delivering 1 J/m^2^/s. γ-ray irradiation was performed using a ^137^Cs source at a dose of 50 Gy/min. Cells growing exponentially were plated at appropriate dilutions on rich medium (YPD) and synthetic plates. Survival was determined as the number of cell-forming colonies on YPD at a given dose divided by the number of non-irradiated colonies. We determined HR frequencies by dividing the number of recombinant colonies growing on selective medium by the number of unselected colonies subjected to the same dose of irradiation. The values obtained after irradiation were corrected by subtracting the number of spontaneous recombinants present on the non-irradiated plates.

### Measurement of spontaneous mutation rates

Spontaneous formation of canavanine-resistant colonies was quantified by a fluctuation test based on a minimum of 27 independent cultures of each strain, initiated from approximately 200 cells and grown to saturation [73].

### Survival following DSB formation

Cells were grown overnight in liquid culture containing lactate before plating. Survival following HO-induced DSB was measured as the number of cells growing on galactose-containing medium divided by the number of colonies growing on YPD. The results shown are the average of at least 3 independent experiments.

### Physical analysis of HO-induced SSA and ectopic gene conversion

Cells were grown in YPD until late exponential phase. Cells were then used to inoculate 400 ml of YPLactate. Cultures were grown to a concentration of 5 to 10×10^6^ cells/ml. A 50 ml sample was removed for the 0 hour time-point and then galactose was added to a final concentration of 2%. Incubation was continued and 50 ml samples were removed at given times. Cells were harvested by centrifugation and washed with water. Cell pellets were then frozen at −20°C. DNA was extracted from the thawed cell pellets and digested accordingly. DNA fragments were separated by electrophoresis on 0.8% agarose gels, transferred to nylon membranes and hybridized with a suitable radioactive probe (Ready-prime II, GE Health Care). Blots were analyzed by using a Typhoon 9600 phosphorimager (GE Health Care) and quantified with ImageQuant Software. The amount of product in the ectopic gene conversion system was measured at 10 hours to avoid the over-estimation of *srs2*Δ cells that had completed repair. The checkpoint is turned off after the completion of repair in this system and cells resume growth. This repeatedly distorts the quantification at 24 hours. However, in the SSA system, we quantified the amount of repair at 24 hours because the reaction was far from complete at 10 hours (the first products appear at 6 hours) and the persistence of the checkpoint impedes *srs2*Δ cell growth.

### Measurement of CO rate in the *arg4* ectopic recombination system

We used the system described in [30]. However, we used a colony-PCR assay to detect the CO among *ARG4* recombinant colonies (Escartin F, De Cian A, Coïc E, Gilquin B, Le Cam E, Veaute X, unpublished data). Briefly, we used primers allowing the discrimination of the parental configurations of the *arg4* locus on chromosome VIII from the reciprocal translocation produced by the CO associated with gene conversion.

### Western blot analysis of Rad53 phosphorylation

Cells were harvested during the time-course experiments previously described for the physical analysis of HO-induced SSA and ectopic gene conversion. Protein extracts were prepared by trichloroacetic acid precipitation. Proteins were separated on 10% SDS-PAGE with an acrylamide/bisacrylamide ratio of 30∶0.4, for 2 hours at 150 V and transferred to nitrocellulose membrane. Membrane was incubated overnight with a goat polyclonal antibody raised against the C terminus sequence of Rad53 (Santa Cruz Biotechnology, yC-19) at a 1/1300 dilution in PBS, 0.1% Tween, 5% milk (w/v); then incubated for 1 hour with secondary horseradish peroxidase-conjugated anti-goat antibody (Santa Cruz Biotechnology, Sc-2020) at a 1/5000 dilution in the same buffer. The blot was revealed by chemiluminescence (ECL Plus, GE Healthcare).

### ChIP experiments and quantitative PCR analyses

Samples were collected during the same time-course experiment performed to monitor the physical analysis of HO-induced SSA (see below). ChIP was carried out as previously described with minor modifications [74]. Samples were incubated with 2 µg of rabbit anti-RPA polyclonal antibody (a gift from V. Géli), of mouse anti-FLAG monoclonal antibody (Sigma) or of rabbit anti-Rad51 polyclonal antibody (Santa Cruz Biotechnology). 50 µl of Magnetic Dynabeads Protein A (Invitrogen) was added to each sample when treated with rabbit antibodies and 50 µl of Magnetic Dynabeads Pan mouse otherwise. After washes, elution of the proteins and reversal of crosslink, samples were treated with proteinase K followed by purification of the DNA with QIAquick PCR purification kit (Qiagen). Quantitative PCR reactions of 180 bp fragments at 0.6 kb or 7.6 kb proximal to the DSB site and at the *ARG5,6* locus were performed using Platinum SYBR Green qPCR SuperMix-UDG (Invitrogen) on an Eppendorf Realplex system.

### Co-immunoprecipitation

Yeast cells were grown in YPD medium to a concentration of 2.5×10^6^ cells/ml. Cells were harvested and washed twice with PBS. Extracts were prepared as previously described [75] without DNAse treatment. The whole cell extract (1 mg) was incubated for 1 hour at 4°C, either with a rabbit anti-Rad52 polyclonal antibody (a gift from S. Jentsch's lab), or with 1 µg of a rabbit anti-Rad51 polyclonal antibody (Santa Cruz Biotechnology). Then, 50 µl of Dynabeads coupled to Protein A (Invitrogen) was added, and the incubation was continued for another hour. The immunoprecipitates were washed twice with 1 ml of lysis buffer and resuspended in 30 µl of Laemmli buffer. The eluted proteins were analyzed by western blot. Proteins were separated on 10% SDS-PAGE and transferred to Hybond-C super membrane (Amersham Biosciences). Proteins were detected with rabbit anti-Rad52 polyclonal antibody (1/2000), rabbit anti-RPA polyclonal antibody (a gift from V. Géli, 1/2500), mouse anti-MYC monoclonal antibody (Sigma, 1/1000), mouse anti-FLAG monoclonal antibody (Sigma, 1/10000) and rabbit anti-Rad51 polyclonal antibody (1/2000). Blots were then incubated with a secondary antibody: horseradish peroxidase-conjugated goat anti-mouse antibody or horseradish-peroxidase-conjugated goat anti-rabbit antibody (GE Healthcare 1/10000). Protein-antibody complexes were visualized by enhanced chemiluminescence using the GE Healthcare ECL Plus system.

### Protein purification

RPA was purified from the protease-deficient yeast stain BJ5496 (*ura3-52*, *trp1*, *leu2Δ1*, *his3Δ200*, *pep4::HIS3*, *prbΔ1.6R*, *can1*). Cells were transformed with three plasmids containing the *RFA1*, *RFA2*, or *RFA3* ORF under the control of a GAL promoter (a gift from R. Kolodner). The RPA heterotrimer was purified as described [76]. Rad51 was overexpressed in *E. coli* BL21 (DE3) pLysS cells transformed with the pEZ5139 plasmid (provided by S. Kowalczykowski) and then purified as described previously [77]. Rad52 and Rad52-L264P were purified from BRL (DE3) pLysS cells transformed with the pET15b-Rad52 or pET15b-Rad52-L264P plasmid. Cells were grown in 8-liters of LB broth containing 100 µg/ml ampicillin at 37°C until A_600_ = 0.8. Protein expression was induced by addition of 0.1 mM IPTG followed by incubation at 30°C for 3 h. Cell lysis was carried out in 50 mM MES (pH 6.5), 450 mM NaCl, 1 mM DTT, 1 mM EDTA, 10% glycerol, 1 mM AEBSF, 10 mM Benzamidine and 2 mM Pepstatin by sonication. Proteins were purified as described previously [78] until the hydroxyapatite column step. Fractions containing Rad52 or Rad52-L264P were pooled and precipitated with 0.45 g/ml ammonium sulfate. Pellets were suspended in 20 mM Tris HCl pH 7.5, 1 M NaCl, 1 mM DTT, 1 mM EDTA and 10% glycerol and then loaded onto Superdex 200 columns (24 ml). Peak fractions were diluted 20 times to obtain a final concentration of 50 mM NaCl and then loaded onto Resource S columns (1 ml). Fractions containing purified Rad52 or Rad52-L264P were pooled, diluted to a final concentration of 200 mM NaCl and finally concentrated using Amicon Ultra 3000 ultrafiltration devices (Millipore). Rad52 and Rad52-L264P concentrations were determined using an extinction coefficient of 2.43×10^4^ at 280 nm.

### Electrophoretic mobility shift assay

Increasing amounts of Rad52 or Rad52-L264P (Figure 7B) were incubated with 0.27 µM 5′ end-Cy5-labeled XV2 oligonucleotide (5′-TGG GTG AAC CTG CAG GTG GGC AAA GAT GTC CTA GCA ATG TAA TCG TCA AGC TTT ATG CCG TT-3′) in buffer E (10 mM Tris-HCl pH 8, 5 mM MgCl_2_, 100 mM NaCl) at 37°C for 10 min. Complexes were separated on 8% native polyacrylamide gels.

### DNA annealing

Primary DNA annealing reactions (Figure 7C) were carried out using the same Cy5-labeled XV2 oligonucleotide as for the electrophoretic mobility shift assay and a reverse-complement oligonucleotide (XV98). Each primer (340 nM nucleotides) was resuspended in buffer E and then mixed (time 0). The annealing reaction was started by adding different concentrations of Rad52 or Rad52-L264P (final volume: 50 µl) and incubation at 25°C. An aliquot of 9 µl was collected every two minutes, transferred into 6 µl of stop buffer (20 µM unlabeled XV2, 0.5% SDS, 0.5 mg/ml Proteinase K) and incubated at 25°C for another 5 min. The effect of RPA and Rad51 on the reaction (Figure 7D and S4) was investigated at 30°C with primers 25 and 26 and the buffers previously described in [7]. RPA or Rad51 (or only storage buffer for control reactions) were incubated with the individual primers for 5 min before mixing the nucleoprotein complexes to start the reaction. All samples were separated on 8% native TBE polyacrylamide gels. Fluorescent signals were revealed with a Typhoon 9400 scanner and quantified with ImageQuant (Molecular Dynamics).

### DNA strand exchange reaction

33 µM (nucleotides) viral (+) strand of φX174 DNA were coated first with 1.1 µM RPA by incubation in SEB buffer (42 mM MOPS pH 7.2, 3 mM Mg acetate, 1 mM DTT, 20 mM NaCl, 25 µg/ml BSA and 2.5 mM ATP) in a final volume of 12.5 µl at 37°C for 5 min. Rad51 filament formation was initiated by adding 5.5 µM Rad51 and different amounts of Rad52 or Rad52-L264P (Figure 7D), or storage buffers as controls. Reactions were incubated at 37°C for 15 min. The addition of 33 µM (nucleotides) of PstI-linearized φX174 dsDNA and 4 mM spermidine initiated the strand exchange reaction. After incubation at 37°C for 90 min, samples were deproteinized by addition of 2 µl of 10 mg/ml Proteinase K, 5% SDS solution at 37°C for 10 min and analyzed by electrophoresis (0.8% agarose gels in 1× TAE buffer). Gels were stained with ethidium bromide and protein bands quantified with ImageQuant (Molecular Dynamics).

### Purification of the 400 nt-long Cy5-labeled ssDNA fragment used for the salt titration analysis

A 5′-biotinylated 400 bp dsDNA fragment was prepared by PCR using the pBR322 plasmid as template. PCR products were loaded onto HiTrap Streptavidin HP columns (GE Healthcare). The non-biotinylated Cy5-labeled strand was purified by elution with 60 mM NaOH.

### Salt titration of protein-DNA complexes

Salt titration of protein-DNA complex formation was performed by first incubating 82.5 nM RPA (1/30 nt) with 2.5 µM ssDNA (5′ end-Cy5-labeled 400 nt-long fragment) in SEB buffer in a final volume reaction of 10 µl at 37°C for 5 min. Increasing concentrations of NaCl were added (Figure 7E), followed by addition of 0.83 µM Rad51 (1/3 nt) and 90.5 nM Rad52 or Rad52-L264P (1/27 nt). After 15 min incubation at 37°C, reactions were stopped with 0.25% glutaraldehyde. 4 µl of 40% sucrose was added to facilitate loading on agarose gel. Nucleoprotein electrophoresis was carried out using 0.5% agarose gels in 1X TAE buffer for 1.5 hours at 150 mA. Fluorescent signals were revealed with a Typhoon 9400 scanners and quantified with ImageQuant (Molecular Dynamics). Western blot analysis was performed after washing the gels twice with transfer buffer (25 mM Tris-HCl, 0.2 M glycine, 0.015% SDS) for 20 min. Proteins were then transferred to PVDF membranes with a semi-dried blotter (Biorad) at 0.8 mA/cm^2^ for 1.25 hours. Membranes were saturated with PBS, 0.1% Tween, 5% milk for 1 hour. Hybridizations with anti-Rad51, anti-Rad52 or anti-RPA antibodies were performed as described for co-immunoprecipitation experiments. Salt titrations of the protein-DNA complex stability were performed as above, but by first incubating proteins and ssDNA in the presence of 60 mM NaCl at 37°C for 15 min to allow the formation of protein-DNA complexes. Additional NaCl was then added to the indicated final concentrations (Figure S5) and the protein-DNA complexes were incubated at 37°C for another 30 min. Reactions were fixed with 0.25% glutaraldehyde. Nucleoprotein gel electrophoresis and western blot analysis were performed as before.

### Electron microscopy analysis

For transmission electron microscopy studies, a fraction of the complex formation reactions was handled as previously described [31]. Positive staining images were taken in order to monitor filament dynamics and formation, whereas negative staining images were taken to obtain structural information on the position of Rad52 (along or at the end of the filaments).

### Western blot analysis of Rad52 sumoylation

Rad52 sumoylation was induced in exponential phase (5×10^6^ cells/ml) by the addition of 0.3% of MMS for 3 hours at 30°C. Rad52 proteins were detected by western blotting as described [11] using a rabbit anti-Rad52 polyclonal antibody at 1/2000 dilution (from S. Jentsch's lab).

### Ni-NTA pull-down of sumoylated Rad52

Rad52-FLAG cells over-expressing *His_7_-SMT3* were collected in exponential phase (2.5×10^6^ cells/ml). Lysates and Ni-NTA pull-Down of sumoylated proteins were carried out according to [63]. Rad52-FLAG was detected with a mouse anti-FLAG monoclonal antibody at 1/10000 dilution (Sigma) on western blots.

### Cycloheximide expression shut-off experiment

Strains were grown to 2.5×10^6^ cells/ml. Expression was shut-off by addition of cycloheximide to a final concentration of 50 µg/ml. For each time-point, 2 ml samples of yeast cells were harvested and protein extracts were prepared [79]. Rad52-FLAG was detected on western blots with a mouse anti-FLAG monoclonal antibody at 1/10000 dilution (Sigma).

## Supporting Information

Figure S1 *rad52-L264P* does not affect cell growth or mutagenesis. (A) Growth of haploid strains on rich medium (YPD) incubated at indicated temperatures. (B) Quantification of spontaneous mutagenesis by measurement of the forward mutation rate in the *CAN1* locus. The results shown are the average of at least 3 independent experiments.
(EPS)

Figure S2 Rad52-L264P interacts with Rad59. Rad52 or Rad52-L264P were immunoprecipitated with a rabbit anti-Rad52 polyclonal antibody from 1 mg of whole cell extracts (without DNase treatment) prepared from *RAD52*, *rad52-L264P* or *rad52*Δ strains that express Rad59-MYC. Proteins from input (50 µg of whole extracts) and from the immunoprecipitated fractions were separated by SDS-PAGE electrophoresis and immunoblotted with a rabbit anti-Rad52 polyclonal antibody or a mouse anti-MYC monoclonal antibody.
(EPS)

Figure S3 Western blot analysis of RPA, Rad52-FLAG and Rad51 protein levels after HO induction in the SSA system. The HO endonuclease was induced in WT cells that express Rad52-FLAG to create a single DSB that can be repaired by SSA. Samples were taken before induction and at 1, 2, 4, 6 and 8 hours after galactose addition. Proteins were extracted by trichloroacetic acid precipitation. 20 µg of proteins were separated by SDS-PAGE and transferred to nitrocellulose membranes. Hybridization with anti-RPA, anti-FLAG and anti-Rad51 antibodies was performed as described in Materials and Methods. Dps1 (loading control) was detected with an anti-Dps1 antibody (1/30000 dilution; a gift from S. Marcand). HO cut triggers a 5-fold increase of Rad51 protein level (6 hours after galactose addition), whereas RPA (only Rfa1 is shown) and Rad52 protein levels are unchanged.
(EPS)

Figure S4 Rad52 and Rad52-L264P annealing activities are similarly inhibited by RPA and Rad51. Reactions were conducted with primers 25 and 26 at 30°C (200 nM each, see Material and Methods) and are illustrated schematically at the top of the panels. The left panel shows a representative gel of DNA annealing reactions and the right panel the quantification of the results. The reactions contained 134 nM Rad51 with or without 30 nM RPA (blue dotted curve), 40 nM Rad52 or Rad52-L264P alone (red for the mutant protein and black solid curve for WT). Red or black dotted curves represent WT or mutant Rad52 with Rad51 and/or RPA. The results are the average of at least three independent experiments and the error bars represent the standard deviation. (A) Rad51 nucleoproteic filament prevents Rad52 and Rad52-L264P annealing with similar efficiency. (B) Same as (A) but the two primers were coated with Rad51. (C) Free Rad51 inhibits equally Rad52 and Rad52-L264P annealing activity.
(EPS)

Figure S5 Salt titration of Rad51-Rad52/Rad52-L264P-ssDNA complex formation and stability. (A) Analysis of the different protein-ssDNA complexes formed. The nucleoprotein complexes were assembled as described in the Material and Methods section in the presence of 60 mM NaCl. RPA (82.5 nM) and Rad51 (0.83 µM) concentrations were chosen based on the strongest inhibitory effect of pre-bound RPA on Rad51 strand exchange activity [80] (line 2). Similarly, we tested different concentrations of Rad52 and found that the optimal stoichiometric ratio between Rad52 and ssDNA corresponded to 1 monomer of Rad52 to 27 nucleotides (90.5 nM) (line 12). This concentration was also found for optimal strand exchange [80]. The unique shifted band observed was absent from the controls (line 2–11). Western blot analysis using antibodies against Rad51 and RPA showed that this shift was related to the binding of Rad51 but not of RPA, confirming that the reaction is optimal in these conditions. Protein-ssDNA complexes were also analyzed by transmission electron microscopy (positive staining). The average numbers of complexes found (and standard deviations) in different controls as well as in the complete reaction with two different salt concentrations are shown in the Table below. 100 molecules were examined for each experiment. We found that Rad51 incubation with RPA-free ssDNA resulted in the formation of incomplete Rad51 filaments. Concomitant addition of Rad52 did not help the formation of complete Rad51 filaments, suggesting that RPA is necessary for complete Rad51 filament formation. However, Rad51 filament formation was clearly inhibited by pre-bound RPA on ssDNA because only 12.5% of Rad51 filaments were formed in the presence of RPA. However, they were all complete Rad51 filaments, indicating that RPA favors the completion of Rad51 filaments that were successfully initiated, possibly by eliminating secondary structures. The concomitant addition of Rad52 and Rad51 on RPA-ssDNA complexes resulted in the formation of 48% of complete Rad51 filaments. These filaments can be associated with Rad52 as observed by western blotting and electron microscopy. (B) Salt titration of the protein-DNA complex stability. Reactions were performed as above to allow the optimal formation of protein-DNA complexes. NaCl concentrations were subsequently increased and the protein-DNA complexes incubated for another 30 min at 37°C. The reactions were fixed with 0.25% glutaraldehyde. Nucleoprotein gel electrophoresis and western blot analysis were performed as described in the Materials and Methods section. (C) Same as (B) except that the salt concentrations were chosen to investigate more precisely the stability of Rad52 on nucleoprotein filaments. All measurements were done in triplicate and the error bars represent the standard error.
(TIF)

Figure S6 Western blot analysis of Rad52-SUMO from total cell extracts. Protein extracts from cells with the indicated genotype were loaded on a 4–12% Bis-Tris gel and immunoblotted with an anti-Rad52 polyclonal antibody. The asterisk denotes a cross-reactive protein. The proportion of sumoylated Rad52 relative to total Rad52 is shown. The specificity of the bands related to sumoylation was checked in *rad52*Δ and *rad52-3KR* strains. Note that the sumoylation of the Rad52-L264P protein induced by 0.3% MMS is reduced two-fold. This can be attributed to a modification of the interaction between the mutated protein and Siz2.
(EPS)

Figure S7 Rad52-3KR sensitivity to proteasome degradation is not suppressed by the *rad52-L264P* mutation. Cycloheximide expression shut-off experiments were performed in a *srs2*Δ background to measure the stability of Rad52-FLAG, Rad52-L264P-FLAG, Rad52-3KR-FLAG and Rad52-L264P-3KR-FLAG proteins. For each time-point, the proteins from whole cell extracts (30 µg at time 0) were separated by SDS-PAGE and immunoblotted with mouse anti-FLAG monoclonal antibody (1/10000). The average of quantification of 3 experiments is shown.
(EPS)

Figure S8 Western blot analysis of the protein encoded by the fusion between the *RAD52* and *SMT3* genes. Protein extracts were immunoblotted with a rabbit anti-Rad52 polyclonal antibody (1/2000). Protein extracts from MMS-induced WT and *RAD52* deleted cells are shown as controls.
(EPS)

Table S1 Doubling time of cells bearing mutations synthetically lethal with *srs2*Δ in the *srs2*Δ *rad52-L264P* background.
(DOCX)

Table S2 Yeast strains used in this study. Strains are grouped according to their background, indicated on the first line of each group. Only differences from this genotype are noted subsequently.
(DOCX)

## References

[1] Cerbinskaite A, Mukhopadhyay A, Plummer ER, Curtin NJ, Edmondson RJ (2011) Defective homologous recombination in human cancers. Cancer Treat Rev 38: 89–100. 21715099 10.1016/j.ctrv.2011.04.015

[2] Pâques F, Haber JE (1999) Multiple pathways of recombination induced by double-strand breaks in *Saccharomyces cerevisiae* . Microbiol Mol Biol Rev 63: 349–404. 10357855 10.1128/mmbr.63.2.349-404.1999 PMC98970

[3] Krogh BO, Symington LS (2004) Recombination proteins in yeast. Annu Rev Genet 38: 233–271. 15568977 10.1146/annurev.genet.38.072902.091500

[4] San Filippo J, Sung P, Klein H (2008) Mechanism of eukaryotic homologous recombination. Annu Rev Biochem 77: 229–257. 18275380 10.1146/annurev.biochem.77.061306.125255

[5] Mortensen UH, Bendixen C, Sunjevaric I, Rothstein R (1996) DNA strand annealing is promoted by the yeast Rad52 protein. Proc Natl Acad Sci U S A 93: 10729–10734. 8855248 10.1073/pnas.93.20.10729 PMC38223

[6] Sugawara N, Haber JE (1992) Characterization of double-strand break-induced recombination: homology requirements and single-stranded DNA formation. Mol Cell Biol 12: 563–575. 1732731 10.1128/mcb.12.2.563-575.1992 PMC364230

[7] Wu Y, Kantake N, Sugiyama T, Kowalczykowski SC (2008) Rad51 protein controls Rad52-mediated DNA annealing. J Biol Chem 283: 14883–14892. 18337252 10.1074/jbc.M801097200 PMC2386943

[8] Lao JP, Oh SD, Shinohara M, Shinohara A, Hunter N (2008) Rad52 promotes postinvasion steps of meiotic double-strand-break repair. Mol Cell 29: 517–524. 18313389 10.1016/j.molcel.2007.12.014 PMC2351957

[9] Shi I, Hallwyl SC, Seong C, Mortensen U, Rothstein R, et al (2009) Role of the Rad52 amino-terminal DNA binding activity in DNA strand capture in homologous recombination. J Biol Chem 284: 33275–33284. 19812039 10.1074/jbc.M109.057752 PMC2785170

[10] Antunez de Mayolo A, Lisby M, Erdeniz N, Thybo T, Mortensen UH, et al (2006) Multiple start codons and phosphorylation result in discrete Rad52 protein species. Nucleic Acids Res 34: 2587–2597. 16707661 10.1093/nar/gkl280 PMC1463902

[11] Sacher M, Pfander B, Hoege C, Jentsch S (2006) Control of Rad52 recombination activity by double-strand break-induced SUMO modification. Nat Cell Biol 8: 1284–1290. 17013376 10.1038/ncb1488

[12] Altmannova V, Eckert-Boulet N, Arneric M, Kolesar P, Chaloupkova R, et al (2010) Rad52 SUMOylation affects the efficiency of the DNA repair. Nucleic Acids Res 38: 4708–4721. 20371517 10.1093/nar/gkq195 PMC2919706

[13] Ohuchi T, Seki M, Branzei D, Maeda D, Ui A, et al (2008) Rad52 sumoylation and its involvement in the efficient induction of homologous recombination. DNA Repair (Amst) 7: 879–889. 18396468 10.1016/j.dnarep.2008.02.005

[14] Torres-Rosell J, Sunjevaric I, De Piccoli G, Sacher M, Eckert-Boulet N, et al (2007) The Smc5-Smc6 complex and SUMO modification of Rad52 regulates recombinational repair at the ribosomal gene locus. Nat Cell Biol 9: 923–931. 17643116 10.1038/ncb1619

[15] Psakhye I, Jentsch S (2012) Protein Group Modification and Synergy in the SUMO Pathway as Exemplified in DNA Repair. Cell 151: 807–820. 23122649 10.1016/j.cell.2012.10.021

[16] Bergink S, Ammon T, Kern M, Schermelleh L, Leonhardt H, et al (2013) Role of Cdc48/p97 as a SUMO-targeted segregase curbing Rad51-Rad52 interaction. Nat Cell Biol 15: 526–532. 23624404 10.1038/ncb2729

[17] Symington LS, Heyer WD (2006) Some disassembly required: role of DNA translocases in the disruption of recombination intermediates and dead-end complexes. Genes Dev 20: 2479–2486. 16980577 10.1101/gad.1477106

[18] Aboussekhra A, Chanet R, Adjiri A, Fabre F (1992) Semidominant suppressors of Srs2 helicase mutations of *Saccharomyces cerevisiae* map in the *RAD51* gene, whose sequence predicts a protein with similarities to procaryotic RecA proteins. Mol Cell Biol 12: 3224–3234. 1620127 10.1128/mcb.12.7.3224-3234.1992 PMC364537

[19] Chanet R, Heude M, Adjiri A, Maloisel L, Fabre F (1996) Semidominant mutations in the yeast Rad51 protein and their relationships with the Srs2 helicase. Mol Cell Biol 16: 4782–4789. 8756636 10.1128/mcb.16.9.4782 PMC231479

[20] Milne GT, Ho T, Weaver DT (1995) Modulation of *Saccharomyces cerevisiae* DNA double-strand break repair by *SRS2* and *RAD51* . Genetics 139: 1189–1199. 7768432 10.1093/genetics/139.3.1189 PMC1206449

[21] Gangloff S, Soustelle C, Fabre F (2000) Homologous recombination is responsible for cell death in the absence of the Sgs1 and Srs2 helicases. Nat Genet 25: 192–194. 10835635 10.1038/76055

[22] Palladino F, Klein HL (1992) Analysis of mitotic and meiotic defects in *Saccharomyces cerevisiae SRS2* DNA helicase mutants. Genetics 132: 23–37. 1327956 10.1093/genetics/132.1.23 PMC1205121

[23] Xu H, Boone C, Klein HL (2004) Mrc1 is required for sister chromatid cohesion to aid in recombination repair of spontaneous damage. Mol Cell Biol 24: 7082–7090. 15282308 10.1128/MCB.24.16.7082-7090.2004 PMC479732

[24] Veaute X, Jeusset J, Soustelle C, Kowalczykowski SC, Le Cam E, et al (2003) The Srs2 helicase prevents recombination by disrupting Rad51 nucleoprotein filaments. Nature 423: 309–312. 12748645 10.1038/nature01585

[25] Krejci L, Van Komen S, Li Y, Villemain J, Reddy MS, et al (2003) DNA helicase Srs2 disrupts the Rad51 presynaptic filament. Nature 423: 305–309. 12748644 10.1038/nature01577

[26] Burgess RC, Lisby M, Altmannova V, Krejci L, Sung P, et al (2009) Localization of recombination proteins and Srs2 reveals anti-recombinase function *in vivo* . J Cell Biol 185: 969–981. 19506039 10.1083/jcb.200810055 PMC2711611

[27] Klein HL (2001) Mutations in recombinational repair and in checkpoint control genes suppress the lethal combination of *srs2*Δ with other DNA repair genes in *Saccharomyces cerevisiae* . Genetics 157: 557–565. 11156978 10.1093/genetics/157.2.557 PMC1461529

[28] Vaze MB, Pellicioli A, Lee SE, Ira G, Liberi G, et al (2002) Recovery from checkpoint-mediated arrest after repair of a double-strand break requires Srs2 helicase. Mol Cell 10: 373–385. 12191482 10.1016/s1097-2765(02)00593-2

[29] Ira G, Malkova A, Liberi G, Foiani M, Haber JE (2003) Srs2 and Sgs1-Top3 suppress crossovers during double-strand break repair in yeast. Cell 115: 401–411. 14622595 10.1016/s0092-8674(03)00886-9 PMC4493758

[30] Robert T, Dervins D, Fabre F, Gangloff S (2006) Mrc1 and Srs2 are major actors in the regulation of spontaneous crossover. EMBO J 25: 2837–2846. 16724109 10.1038/sj.emboj.7601158 PMC1500851

[31] Dupaigne P, Le Breton C, Fabre F, Gangloff S, Le Cam E, et al (2008) The Srs2 helicase activity is stimulated by Rad51 filaments on dsDNA: implications for crossover incidence during mitotic recombination. Mol Cell 29: 243–254. 18243118 10.1016/j.molcel.2007.11.033

[32] Mitchel K, Lehner K, Jinks-Robertson S (2013) Heteroduplex DNA position defines the roles of the Sgs1, Srs2, and Mph1 helicases in promoting distinct recombination outcomes. PLoS Genet 9: e1003340. 23516370 10.1371/journal.pgen.1003340 PMC3597516

[33] Aboussekhra A, Chanet R, Zgaga Z, Cassier-Chauvat C, Heude M, et al (1989) *RADH*, a gene of *Saccharomyces cerevisiae* encoding a putative DNA helicase involved in DNA repair. Characteristics of *radH* mutants and sequence of the gene. Nucleic Acids Res 17: 7211–7219. 2552405 10.1093/nar/17.18.7211 PMC334801

[34] Mortensen UH, Erdeniz N, Feng Q, Rothstein R (2002) A molecular genetic dissection of the evolutionarily conserved N terminus of yeast Rad52. Genetics 161: 549–562. 12072453 10.1093/genetics/161.2.549 PMC1462154

[35] Shinohara A, Shinohara M, Ohta T, Matsuda S, Ogawa T (1998) Rad52 forms ring structures and co-operates with RPA in single-strand DNA annealing. Genes Cells 3: 145–156. 9619627 10.1046/j.1365-2443.1998.00176.x

[36] Plate I, Hallwyl SC, Shi I, Krejci L, Muller C, et al (2008) Interaction with RPA is necessary for Rad52 repair center formation and for its mediator activity. J Biol Chem 283: 29077–29085. 18703507 10.1074/jbc.M804881200 PMC2570898

[37] Rong L, Palladino F, Aguilera A, Klein HL (1991) The hyper-gene conversion *hpr5-1* mutation of *Saccharomyces cerevisiae* is an allele of the *SRS2/RADH* gene. Genetics 127: 75–85. 1849857 10.1093/genetics/127.1.75 PMC1204314

[38] Schild D (1995) Suppression of a new allele of the yeast *RAD52* gene by overexpression of *RAD51*, mutations in *srs2* and *ccr4*, or mating-type heterozygosity. Genetics 140: 115–127. 7635279 10.1093/genetics/140.1.115 PMC1206541

[39] Schmidt KH, Kolodner RD (2006) Suppression of spontaneous genome rearrangements in yeast DNA helicase mutants. Proc Natl Acad Sci U S A 103: 18196–18201. 17114288 10.1073/pnas.0608566103 PMC1838729

[40] Fabre F, Chan A, Heyer WD, Gangloff S (2002) Alternate pathways involving Sgs1/Top3, Mus81/Mms4, and Srs2 prevent formation of toxic recombination intermediates from single-stranded gaps created by DNA replication. Proc Natl Acad Sci U S A 99: 16887–16892. 12475932 10.1073/pnas.252652399 PMC139239

[41] Yeung M, Durocher D (2011) Srs2 enables checkpoint recovery by promoting disassembly of DNA damage foci from chromatin. DNA Repair (Amst) 10: 1213–1222. 21982442 10.1016/j.dnarep.2011.09.005

[42] Sugiyama T, New JH, Kowalczykowski SC (1998) DNA annealing by Rad52 protein is stimulated by specific interaction with the complex of replication protein A and single-stranded DNA. Proc Natl Acad Sci U S A 95: 6049–6054. 9600915 10.1073/pnas.95.11.6049 PMC27583

[43] Song B, Sung P (2000) Functional interactions among yeast Rad51 recombinase, Rad52 mediator, and replication protein A in DNA strand exchange. J Biol Chem 275: 15895–15904. 10748203 10.1074/jbc.M910244199

[44] Davis AP, Symington LS (2001) The yeast recombinational repair protein Rad59 interacts with Rad52 and stimulates single-strand annealing. Genetics 159: 515–525. 11606529 10.1093/genetics/159.2.515 PMC1461847

[45] Cortes-Ledesma F, Malagon F, Aguilera A (2004) A novel yeast mutation, *rad52-L89F*, causes a specific defect in Rad51-independent recombination that correlates with a reduced ability of Rad52-L89F to interact with Rad59. Genetics 168: 553–557. 15454565 10.1534/genetics.104.030551 PMC1448092

[46] Johnson ES (2004) Protein modification by SUMO. Annu Rev Biochem 73: 355–382. 15189146 10.1146/annurev.biochem.73.011303.074118

[47] Lettier G, Feng Q, de Mayolo AA, Erdeniz N, Reid RJ, et al (2006) The role of DNA double-strand breaks in spontaneous homologous recombination in *S. cerevisiae* . PLoS Genet 2: e194. 17096599 10.1371/journal.pgen.0020194 PMC1635536

[48] Bai Y, Davis AP, Symington LS (1999) A novel allele of *RAD52* that causes severe DNA repair and recombination deficiencies only in the absence of *RAD51* or *RAD59* . Genetics 153: 1117–1130. 10545446 10.1093/genetics/153.3.1117 PMC1460819

[49] Boundy-Mills KL, Livingston DM (1993) A *Saccharomyces cerevisiae RAD52* allele expressing a C-terminal truncation protein: activities and intragenic complementation of missense mutations. Genetics 133: 39–49. 8417987 10.1093/genetics/133.1.39 PMC1205296

[50] Krejci L, Song B, Bussen W, Rothstein R, Mortensen UH, et al (2002) Interaction with Rad51 is indispensable for recombination mediator function of Rad52. J Biol Chem 277: 40132–40141. 12171935 10.1074/jbc.M206511200

[51] de Mayolo AA, Sunjevaric I, Reid R, Mortensen UH, Rothstein R, et al (2010) The *rad52-Y66A* allele alters the choice of donor template during spontaneous chromosomal recombination. DNA Repair (Amst) 9: 23–32. 19892607 10.1016/j.dnarep.2009.10.001 PMC2818265

[52] Saponaro M, Callahan D, Zheng X, Krejci L, Haber JE, et al (2010) Cdk1 targets Srs2 to complete synthesis-dependent strand annealing and to promote recombinational repair. PLoS Genet 6: e1000858. 20195513 10.1371/journal.pgen.1000858 PMC2829061

[53] Lee SE, Moore JK, Holmes A, Umezu K, Kolodner RD, et al (1998) *Saccharomyces* Ku70, mre11/rad50 and RPA proteins regulate adaptation to G2/M arrest after DNA damage. Cell 94: 399–409. 9708741 10.1016/s0092-8674(00)81482-8

[54] Katou Y, Kanoh Y, Bando M, Noguchi H, Tanaka H, et al (2003) S-phase checkpoint proteins Tof1 and Mrc1 form a stable replication-pausing complex. Nature 424: 1078–1083. 12944972 10.1038/nature01900

[55] Symington LS (2002) Role of *RAD52* epistasis group genes in homologous recombination and double-strand break repair. Microbiol Mol Biol Rev 66: 630–70. 12456786 10.1128/MMBR.66.4.630-670.2002 PMC134659

[56] Li X, Heyer WD (2009) Rad54 controls access to the invading 3′-OH end after Rad51-mediated DNA strand invasion in homologous recombination in *Saccharomyces cerevisiae* . Nucleic Acids Res 37: 638–646. 19074197 10.1093/nar/gkn980 PMC2632917

[57] Rossi MJ, Mazin AV (2008) Rad51 protein stimulates the branch migration activity of Rad54 protein. J Biol Chem 283: 24698–24706. 18617519 10.1074/jbc.M800839200 PMC3259846

[58] Sinha M, Peterson CL (2008) A Rad51 presynaptic filament is sufficient to capture nucleosomal homology during recombinational repair of a DNA double-strand break. Mol Cell 30: 803–810. 18570881 10.1016/j.molcel.2008.04.015 PMC4461863

[59] Cejka P, Kowalczykowski SC (2010) The full-length *Saccharomyces cerevisiae* Sgs1 protein is a vigorous DNA helicase that preferentially unwinds holliday junctions. J Biol Chem 285: 8290–8301. 20086270 10.1074/jbc.M109.083196 PMC2832980

[60] Mankouri HW, Ashton TM, Hickson ID (2011) Holliday junction-containing DNA structures persist in cells lacking Sgs1 or Top3 following exposure to DNA damage. Proc Natl Acad Sci U S A 108: 4944–4949. 21383164 10.1073/pnas.1014240108 PMC3064375

[61] Moldovan GL, Dejsuphong D, Petalcorin MI, Hofmann K, Takeda S, et al (2011) Inhibition of Homologous Recombination by the PCNA-Interacting Protein PARI. Mol Cell 45: 75–86. 22153967 10.1016/j.molcel.2011.11.010 PMC3267324

[62] Feng Z, Scott SP, Bussen W, Sharma GG, Guo G, et al (2011) Rad52 inactivation is synthetically lethal with BRCA2 deficiency. Proc Natl Acad Sci U S A 108: 686–691. 21148102 10.1073/pnas.1010959107 PMC3021033

[63] Ulrich HD, Davies AA (2009) *In vivo* detection and characterization of sumoylation targets in *Saccharomyces cerevisiae* . Methods Mol Biol 497: 81–103. 19107412 10.1007/978-1-59745-566-4_6

[64] Goldstein AL, McCusker JH (1999) Three new dominant drug resistance cassettes for gene disruption in *Saccharomyces cerevisiae* . Yeast 15: 1541–1553. 10514571 10.1002/(SICI)1097-0061(199910)15:14<1541::AID-YEA476>3.0.CO;2-K

[65] Ito H, Fukuda Y, Murata K, Kimura A (1983) Transformation of intact yeast cells treated with alkali cations. J Bacteriol 153: 163–168. 6336730 10.1128/jb.153.1.163-168.1983 PMC217353

[66] Longtine MS, McKenzie Ar, Demarini DJ, Shah NG, Wach A, et al (1998) Additional modules for versatile and economical PCR-based gene deletion and modification in *Saccharomyces cerevisiae* . Yeast 14: 953–961. 9717241 10.1002/(SICI)1097-0061(199807)14:10<953::AID-YEA293>3.0.CO;2-U

[67] De Antoni A, Gallwitz D (2000) A novel multi-purpose cassette for repeated integrative epitope tagging of genes in *Saccharomyces cerevisiae* . Gene 246: 179–185. 10767539 10.1016/s0378-1119(00)00083-4

[68] Altschul SF, Madden TL, Schaffer AA, Zhang J, Zhang Z, et al (1997) Gapped BLAST and PSI-BLAST: a new generation of protein database search programs. Nucleic Acids Res 25: 3389–3402. 9254694 10.1093/nar/25.17.3389 PMC146917

[69] Schaffer AA, Aravind L, Madden TL, Shavirin S, Spouge JL, et al (2001) Improving the accuracy of PSI-BLAST protein database searches with composition-based statistics and other refinements. Nucleic Acids Res 29: 2994–3005. 11452024 10.1093/nar/29.14.2994 PMC55814

[70] Edgar RC (2004) MUSCLE: multiple sequence alignment with high accuracy and high throughput. Nucleic Acids Res 32: 1792–1797. 15034147 10.1093/nar/gkh340 PMC390337

[71] Jones DT (1999) Protein secondary structure prediction based on position-specific scoring matrices. J Mol Biol 292: 195–202. 10493868 10.1006/jmbi.1999.3091

[72] Waterhouse AM, Procter JB, Martin DM, Clamp M, Barton GJ (2009) Jalview Version 2–a multiple sequence alignment editor and analysis workbench. Bioinformatics 25: 1189–1191. 19151095 10.1093/bioinformatics/btp033 PMC2672624

[73] Foster PL (2006) Methods for determining spontaneous mutation rates. Methods Enzymol 409: 195–213. 16793403 10.1016/S0076-6879(05)09012-9 PMC2041832

[74] Sugawara N, Haber JE (2006) Repair of DNA double strand breaks: *in vivo* biochemistry. Methods Enzymol 408: 416–429. 16793384 10.1016/S0076-6879(06)08026-8

[75] Strahl-Bolsinger S, Hecht A, Luo K, Grunstein M (1997) Sir2 and Sir4 interactions differ in core and extended telomeric heterochromatin in yeast. Genes Dev 11: 83–93. 9000052 10.1101/gad.11.1.83

[76] Kantake N, Sugiyama T, Kolodner RD, Kowalczykowski SC (2003) The recombination-deficient mutant RPA (*rfa1-t11*) is displaced slowly from single-stranded DNA by Rad51 protein. J Biol Chem 278: 23410–23417. 12697761 10.1074/jbc.M302995200

[77] Zaitseva EM, Zaitsev EN, Kowalczykowski SC (1999) The DNA binding properties of *Saccharomyces cerevisiae* Rad51 protein. J Biol Chem 274: 2907–2915. 9915828 10.1074/jbc.274.5.2907

[78] New JH, Sugiyama T, Zaitseva E, Kowalczykowski SC (1998) Rad52 protein stimulates DNA strand exchange by Rad51 and replication protein A. Nature 391: 407–410. 9450760 10.1038/34950

[79] Belle A, Tanay A, Bitincka L, Shamir R, O'Shea EK (2006) Quantification of protein half-lives in the budding yeast proteome. Proc Natl Acad Sci U S A 103: 13004–13009. 16916930 10.1073/pnas.0605420103 PMC1550773

[80] Sung P (1997) Function of yeast Rad52 protein as a mediator between replication protein A and the Rad51 recombinase. J Biol Chem 272: 28194–28197. 9353267 10.1074/jbc.272.45.28194

