# Supplementary figures and images for: Rad52 Sumoylation Prevents the Toxicity of Unproductive Rad51 Filaments Independently of the Anti-Recombinase Srs2
Source: PLoS Genet. 2013 Oct 10;9(10):e1003833. doi: 10.1371/journal.pgen.1003833 (PMC3794917; doi:10.1371/journal.pgen.1003833)

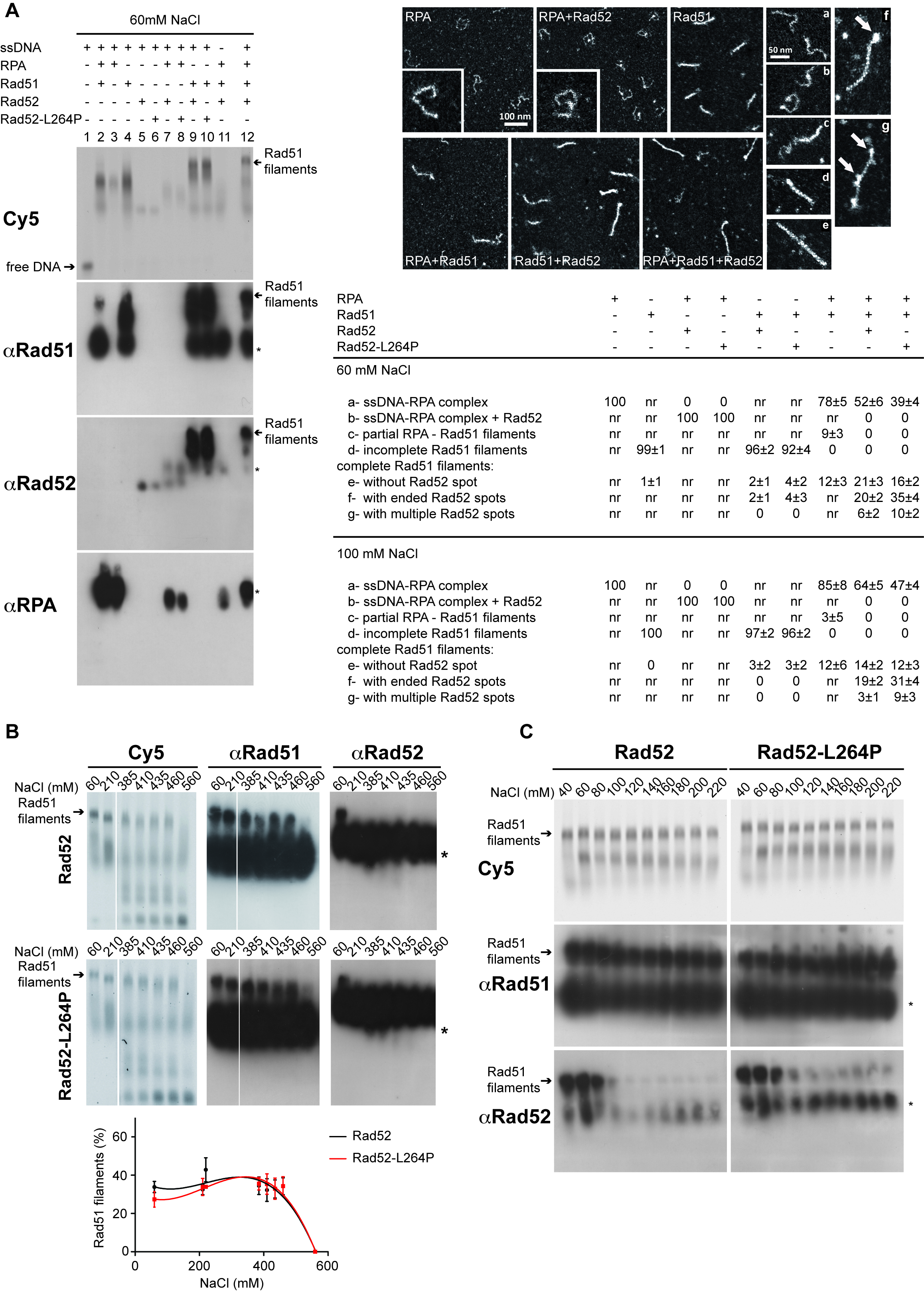

Supplement: Figure S5 — Salt titration of Rad51-Rad52/Rad52-L264P-ssDNA complex formation and stability. (A) Analysis of the different protein-ssDNA complexes formed. The nucleoprotein complexes were assembled as described in the Material and Methods section in the presence of 60 mM NaCl. RPA (82.5 nM) and Rad51 (0.83 µM) concentrations were chosen based on the strongest inhibitory effect of pre-bound RPA on Rad51 strand exchange activity [80] (line 2). Similarly, we tested different concentrations of Rad52 and found that the optimal stoichiometric ratio between Rad52 and ssDNA corresponded to 1 monomer of Rad52 to 27 nucleotides (90.5 nM) (line 12). This concentration was also found for optimal strand exchange [80]. The unique shifted band observed was absent from the controls (line 2–11). Western blot analysis using antibodies against Rad51 and RPA showed that this shift was related to the binding of Rad51 but not of RPA, confirming that the reaction is optimal in these conditions. Protein-ssDNA complexes were also analyzed by transmission electron microscopy (positive staining). The average numbers of complexes found (and standard deviations) in different controls as well as in the complete reaction with two different salt concentrations are shown in the Table below. 100 molecules were examined for each experiment. We found that Rad51 incubation with RPA-free ssDNA resulted in the formation of incomplete Rad51 filaments. Concomitant addition of Rad52 did not help the formation of complete Rad51 filaments, suggesting that RPA is necessary for complete Rad51 filament formation. However, Rad51 filament formation was clearly inhibited by pre-bound RPA on ssDNA because only 12.5% of Rad51 filaments were formed in the presence of RPA. However, they were all complete Rad51 filaments, indicating that RPA favors the completion of Rad51 filaments that were successfully initiated, possibly by eliminating secondary structures. The concomitant addition of Rad52 and Rad51 on RPA-ssDNA complexes resul [file pgen.1003833.s005.tif]
