# Supplementary material for: Rad52 Sumoylation Prevents the Toxicity of Unproductive Rad51 Filaments Independently of the Anti-Recombinase Srs2
Source: PLoS Genet. 2013 Oct 10;9(10):e1003833. doi: 10.1371/journal.pgen.1003833 (PMC3794917; doi:10.1371/journal.pgen.1003833)
Supplement: Table S1 — Doubling time of cells bearing mutations synthetically lethal with srs2Δ in the srs2Δ rad52-L264P background. (DOCX) [file pgen.1003833.s009.docx]

| **Table S1. Doubling time of cells bearing mutations synthetically lethal with *srs2*∆ in the *srs2*∆ *rad52-L264P* background.** | |  |
| --- | --- | --- |
|  | Genotype | Doubling time |
|  | *srs2::LEU2 rad52-L264P* | 91 |
|  | *sgs1::URA3 srs2::LEU2 rad52-L264P* | 92 |
|  | *rad54::LEU2 srs2::LEU2 rad52-L264P* | 105 |
|  | *rad50::URA3 srs2::LEU2 rad52-L264P* | 147 |
|  | *mrc1::NATMX srs2::LEU2 rad52-L264P* | 136 |
|  | *rrm3::NATMX srs2::LEU2 rad52-L264P* | 100 |
|  | *ctf18::NATMX srs2::LEU2 rad52-L264P* | 139 |
