# Supplementary material for: Rad52 Sumoylation Prevents the Toxicity of Unproductive Rad51 Filaments Independently of the Anti-Recombinase Srs2
Source: PLoS Genet. 2013 Oct 10;9(10):e1003833. doi: 10.1371/journal.pgen.1003833 (PMC3794917; doi:10.1371/journal.pgen.1003833)
Supplement: Table S2 — Yeast strains used in this study. Strains are grouped according to their background, indicated on the first line of each group. Only differences from this genotype are noted subsequently. (DOCX) [file pgen.1003833.s010.docx]

| **Table S2. Yeast strains used in this study.** | |  |
| --- | --- | --- |
| Strain | Genotype | Source |
| FF18733 | *MAT***a** *leu2-3, 112 trp1-289 ura3-52 lys1-1 his7-2* | F. Fabre |
| FF18742 | *MAT***a** *rad52::URA3* | F. Fabre |
| FF18744 | *MAT***a** *srs2::LEU2* | F. Fabre |
| FF18964 | *MAT***a** *rad50::URA3* | F. Fabre |
| FF18973 | *MAT***a** *rad54::LEU2* | F. Fabre |
| FF181495 | *MAT***a** *sgs1::URA3* | F. Fabre |
| ECS57 | *MAT***a** *rrm3::NATMX* | This study |
| ECS74 | *MAT***a** *mrc1::NATMX* | This study |
| ECS75 | *MAT***a** *ctf18::NATMX* | This study |
| ECS1914 | *MAT***a** *siz2::KanMX* | This study |
| ECS1921 | *MAT***a** *rad52-L264P* | This study |
| ECS2422 | *MAT***a** *rad52-L264A* | This study |
| FF18746 | *MAT***a** *rad52::URA3 srs2::LEU2* | This study |
| ECS1888 | *MAT*α *rad52-L264P srs2::URA3* | This study |
| ECS1892 | *MAT*α *rad52-L264P srs2::LEU2* | This study |
| ECS2271 | *MAT***a** *rad52-L264P srs2::LEU2* | This study |
| ECS2431 | *MAT***a** *rad52-L264A srs2::LEU2* | This study |
| ECS2146 | *MAT***a** *siz2::KanMX srs2::URA3* | This study |
| ECS2174 | *MAT***a** *rad52-L264P siz2::KanMX* | This study |
| ECS2176 | *MAT***a** *rad52-L264P siz2::KanMX srs2::LEU2* | This study |
| ECS2275 | *MAT***a** pRS426 | This study |
| ECS2277 | *MAT***a** *rad52-L264P* pRS426 | This study |
| ECS1898 | *MAT***a** *srs2::LEU2* pRS426 | This study |
| ECS1938 | *MAT***a** *srs2::LEU2 pRS426-SIZ2 (pEC49)* | This study |
| ECS2299 | *MAT***a** *srs2::LEU2 rad52::URA3 pRS426-SIZ2 (pEC49)* | This study |
| ECS2269 | *MAT***a** *rad52::ura3 ura3:: rad52-3KR::KanMX* | This study |
| ECS2527 | *MAT***a** *rad52::ura3 ura3:: rad52-3KR-L264P::KanMX* | This study |
| ECS2186 | *MAT***a** *rad52::ura3 ura3:: rad52-3KR::KanMX srs2::LEU2* | This study |
| ECS2530 | *MAT***a** *rad52::ura3 ura3:: rad52-3KR-L264P::KanMX srs2::LEU2* | This study |
| ECS2161 | *MAT***a** *ura3:: rad52-3KR::KanMX srs2::LEU2* | This study |
| ECS2301 | *MAT***a** *srs2::LEU2 rad52::ura3 ura3:: rad52-3KR::KanMX pRS426* | This study |
| ECS2303 | *MAT***a** *srs2::LEU2 rad52::ura3 ura3:: rad52-3KR::KanMX pRS426-SIZ2 (pEC49)* | This study |
| ECS2404 | *MAT***a** *RAD59-9MYC-KanMX* | This study |
| ECS2406 | *MAT***a** *rad52-L264P RAD59-9MYC-KanMX* | This study |
| ECS2419 | *MAT***a** *rad52::URA3 RAD59-9MYC-KanMX* | This study |
| AES21 | *MAT***a** *RAD52-*flag*::KanMX* | This study |
| AES23 | *MAT***a** *rad52-L264P-*flag*::KanMX* | This study |
| ECS2764 | *MAT***a** *RAD52-*flag*::KanMX pRS426* | This study |
| ECS2766 | *MAT***a** *RAD52-*flag*::KanMX pRS426-SIZ2 (pEC49)* | This study |
| AES97 | *MAT***a** *RAD52-*flag*::KanMX* Yep181-CUP-His_7_-Smt3 *pRS426* | This study |
| AES99 | *MAT***a** *RAD52-*flag*::KanMX* Yep181-CUP-His_7_-Smt3 *pRS426-SIZ2 (pEC49)* | This study |
| AES101 | *MAT***a** *rad52-L264P-*flag*::KanMX* Yep181-CUP-His_7_-Smt3 *pRS426* | This study |
| ECS2343 | *MAT***a** *RAD52-SMT3::NATMX* | This study |
| ECS2333 | *MAT***a** *RAD52-SMT3::NATMX srs2::LEU2* | This study |
|  |  |  |
| FF18985 | *MAT*α *leu2-3, 112 trp1-289 ura3-52 lys2-1 his7-1* | F. Fabre |
| FF18994 | *MAT*α *srs2::LEU2* | F. Fabre |
| ECS2306 | *MAT*α *rad52-L264P* | This study |
| ECS2307 | *MAT*α *rad52-L264P srs2::LEU2* | This study |
|  |  |  |
| YMV80 | *ade1-100 ura3-52 leu2-3,112 lys5 hml::ADE1 mat::hisG hmr::ADE1 leu2-cs his4::NAT-leu2∆5’ ade3::GAL::HO* | J. Haber |
| ECS2641 | *srs2::KanMX* | This study |
| ECS2687 | *rad52-L264P* | This study |
| EMY222 | *RAD52-SMT3::KANMX* | This study |
| ECS2693 | *rad52-L264P srs2::KanMX* | This study |
| EMY223  EMY110  EMY105  EMY210 | *RAD52-SMT3::KanMX srs2::HPHMX*  *RAD52-flag::KANMX*  *rad52-L264P-flag::KANMX*  *RAD52-SMT3-flag::KANMX* | This study  This study  This study  This study |
|  |  |  |
| tGI354 | *hml::ADE1 MATalpha hmr::ADE1 arg5,6::MATa-inc::HPH1 ade3::GAL::HO* | J. Haber |
| ECS2632 | *srs2::KanMX* | This study |
| ECS2688 | *rad52-L264P* | This study |
| EMY224 | *RAD52-SMT3::KanMX* | This study |
| ECS2690 | *rad52-L264P srs2::KanMX* | This study |
| EMY225 | *RAD52-SMT3::KanMX srs2::NATMX* | This study |

Strains are grouped according to their background, indicated on the first line of each group. Only differences from this genotype are noted subsequently.
